# Krüppel-Like Factor 6 Induces RNA Polymerase II Subunit RPB1 to Promote Kidney Injury

**DOI:** 10.1681/ASN.0000000722

**Published:** 2025-05-06

**Authors:** Sian E. Piret, Samaneh DiMartino, Maanasa Hanubal, Merin Davis, Jiakang Wang, Tej Bahadur, Asha Rath, Nehaben A. Gujarati, Bismark O. Frimpong, Robert Bronstein, Monica P. Revelo, Yiqing Guo, Sandeep K. Mallipattu

**Affiliations:** 1 Division of Nephrology and Hypertension, Department of Medicine, Stony Brook University, Stony Brook, New York; 2 Department of Pathology, University of Utah, Salt Lake City, Utah; 3 Renal Section, Northport VA Medical Center, Northport, New York

**Keywords:** AKI, CKD, fibrosis, proximal tubule

## Abstract

**Key Points:**

- Single nuclear RNA sequencing after DNA damage–induced AKI identified an injured proximal tubule cluster with high *Polr2a* (RNA polymerase subunit B1), an RNA polymerase II subunit.
- *POLR2A* knockdown in injured cells decreased inflammatory and fibrotic gene expression, dedifferentiation, DNA damage, and cell cycle arrest.
- RNA polymerase subunit B1 was higher in mice overexpressing transcription factor Krüppel-like factor 6 and associated with worse injury after DNA damage.

**Background:**

Initial proximal tubule cell injury and dedifferentiation contribute to AKI, and persistent dedifferentiation drives fibrosis and CKD. Proximal tubule–specific knockdown of zinc-finger transcription factor *Krüppel-like factor 6* (*Klf6*) attenuates the AKI to CKD transition. Our aim was to study the early transcriptional mechanisms by which KLF6 induction exacerbates proximal tubular injury and eventual fibrosis.

**Methods:**

Aristolochic acid I–treated wild-type and KLF6 overexpression mice underwent single nucleus (sn)RNA-seq and single nucleus assay for transposase-accessible chromatin sequencing (acute phase) and assessment of kidney function, injury, and fibrosis (remodeling phase). *POLR2A* was knocked down in human kidney cells and cell number, gene expression, differentiation, DNA damage, and cell cycle assessed. Kidney sections from fibrotic mouse models and human CKD secondary to aristolochic acid and diabetes were assessed for RNA polymerase subunit B1 (RPB1) expression.

**Results:**

snRNA-seq identified an injured proximal tubule cluster with high expression of *Klf6* and RNA polymerase II subunit a (*Polr2a*) encoding RPB1. After injury, RPB1-positive cells accumulated and were associated with dedifferentiated proximal tubules. *POLR2A* knockdown in injured cells increased cell death, but reduced inflammatory and fibrotic gene expression, dedifferentiation, DNA damage, and G2/M cell cycle arrest, with a transcriptional switch from long genes to short genes. Single nucleus assay for transposase-accessible chromatin sequencing demonstrated an open chromatin region in *Polr2a* intron 1 in injured proximal tubule cells, containing a KLF6-binding site. Knockdown of *KLF6* reduced *POLR2A* induction, while proximal tubule–specific *KLF6* further increased *Polr2a* levels after injury. Mice with tubule-specific *KLF6* induction had more RPB1-positive proximal tubules and more injury post-AKI. Human kidney samples with DNA damage–induced CKD and diabetic kidney disease also had high *POLR2A*/RPB1 expression in dedifferentiated proximal tubule cells.

**Conclusions:**

Prolonged high expression of RPB1 is associated with dedifferentiated proximal tubule cells. Mice overexpressing *KLF6* had higher expression of RPB1 and worse kidney injury after DNA damage.

## Visual Abstract

**Figure:**
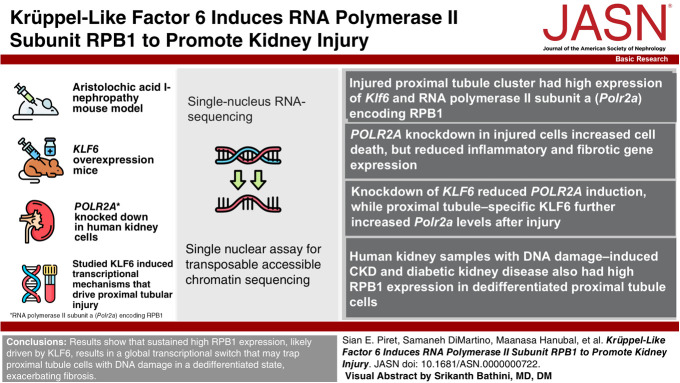

## Introduction

CKD has a prevalence in the US population of approximately 15% and causes significant morbidity and mortality. CKD may develop after AKI, due to ischemia, environmental or medical toxins, or sepsis.^1^ The proximal tubule is particularly susceptible to AKI, due to high metabolic demand and critical role in excretion of toxins. Injured proximal tubule cells are key drivers of the inflammatory and fibrotic processes occurring in AKI.^2^ During injury, proximal tubule cells dedifferentiate and can re-enter the cell cycle, proliferate, and repair tubular structures that are lost through cell death.^3^ Thus, initial proximal tubular dedifferentiation is likely necessary for adaptive repair after AKI. However, a maladaptive dedifferentiation state can result from G2/M cell cycle arrest, and arrested cells promote fibrosis through expression and secretion of factors such as connective tissue growth factor (CTGF) and TGF-*β*.^4–7^ The factors and pathways controlling proximal tubular adaptive versus maladaptive dedifferentiation and redifferentiation after injury are not fully understood.

DNA damage can result directly from medical therapies such as chemotherapeutics (*e.g*., cisplatin) or naturally occurring toxic compounds such as aristolochic acid I (AAI), or indirectly in other types of AKI such as in ischemia-reperfusion injury, due to production of reactive oxygen species.^8^ We previously demonstrated that mice with proximal tubule–specific knockdown of the transcription factor Krüppel-like factor 6 (KLF6), which is induced in the proximal tubule in injury, were protected from AAI-induced AKI and fibrosis.^9^ Furthermore, mice with global human *KLF6* expression (h*KLF6*^OE^ mice) had exacerbated proximal tubule injury and AKI post–AAI treatment. However, *Klf6* is expressed in multiple cell types within the kidney. We therefore sought to determine early proximal tubule–specific pathways driven by KLF6 in DNA damage–induced kidney injury, and the consequences of these early KLF6-induced pathways for the resulting kidney fibrosis.

## Methods

Additional details regarding methods including snRNA-seq, single nucleus assay for transposase-accessible chromatin sequencing (snATAC-seq), and associated analyses, histologic analyses, and cell culture studies are provided in the Supplemental Material.

### Study Approvals

Mouse procedures were approved by the Stony Brook University Institutional Animal Care and Use Committee. National Institutes of Health Guide for the Care and Use of Laboratory Animals was followed. Human AA kidney tissue specimens were fully deidentified and came from a previously published dataset.^10,11^ Deidentified human diabetic kidney disease (DKD) biopsy samples from University of Utah were scored for fibrosis by a renal pathologist (M.P. Revelo). Control kidney biopsy specimens were acquired from the unaffected pole of kidneys that were removed because of renal cell carcinoma. The study was approved by the Stony Brook University Institutional Review Board (798611).

### Mouse Models

Control (wild-type or TRE-h*KLF6*) mice or doxycycline-treated mice with global (CMV-chicken beta-actin–reverse tetracycline-controlled transactivator; TRE-h*KLF6* = h*KLF6*^OE^)^9^ or tubular (*Pax8*-rtTA; TRE-h*KLF6*=h*KLF6*^TOE^) overexpression of human *KLF6* were administered AAI, repeated low-dose cisplatin (RLDC), or underwent unilateral ureteral obstruction (UUO), as previously reported.^9,12,13^

### Cell Culture Studies

Human kidney 2 (HK-2) cells treated with scrambled (control) or *POLR2A* short interfering RNAs and DMSO (vehicle) or 25 *µ*M AAI were used for cell proliferation, immunofluorescence, gene expression, and cell cycle analyses.

### Statistical Analysis

The exact test used for each experiment is denoted in the figure legends. All data are expressed as mean±SEM (mice, cells) or box and whiskers, with box edges as 25th and 75th percentiles, whiskers at minimum and maximum, and line at the median (human data). Analyses were undertaken using GraphPad Prism 9 and significance assumed when *P* < 0.05. Sample sizes for h*KLF6*^TOE^ mice studies were calculated based on estimated effect sizes from our previous studies of *Klf6* knockdown and overexpression.^9^

## Results

### Single Nucleus RNA Sequencing Revealed Two Injured Proximal Tubule Clusters after AAI-Induced AKI

**Figure 1:**
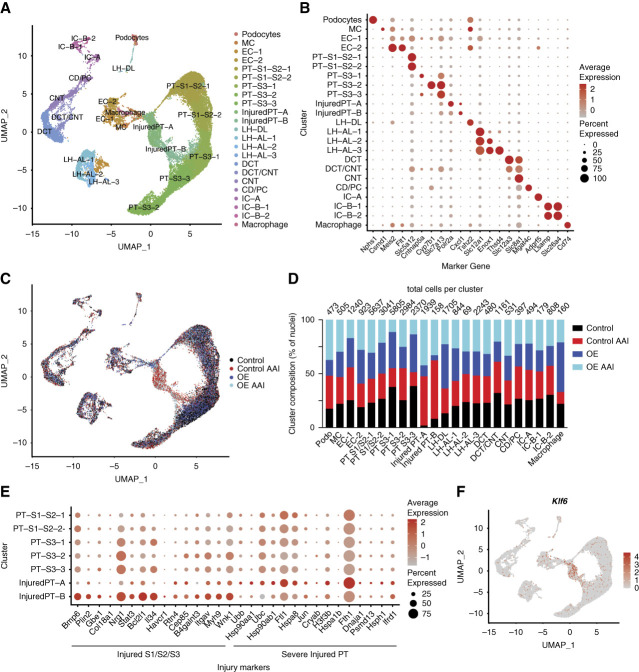
**snRNA-seq of control and h*KLF6***^**OE**^ **mouse kidneys with and without AAI-induced injury.** (A) UMAP plot of the 23 clusters generated by unsupervised clustering of the 34,290 sequenced cells. (B) Cell type–specific marker genes in the 23 clusters. (C) UMAP plot of the 23 clusters colored by the sample of origin. (D) Relative cell type abundance in the four samples. Numbers above the bars indicate the total number of cells in each cluster. (E) Expression of previously reported marker genes from injured S1/S2/S3 and severe injured proximal tubule clusters.^14^ (F) UMAP showing *Klf6* expression levels. AAI, aristolochic acid I; AL, ascending limb; CD/PC, collecting duct principal cell; CNT, connecting tubule; DCT, distal convoluted tubule; DL, descending limb; EC, endothelial cells; IC, intercalated cell; *Klf6*, Krüppel-like factor 6; LH, loop of Henle; MC, mesangial cells; OE, overexpression; PT, proximal tubule; snRNA-seq, single nucleus RNA sequencing; UMAP, uniform manifold approximation and projection.

To generate hypotheses for early KLF6-driven pathways, we undertook single nucleus RNA sequencing (snRNA-seq) in control and global h*KLF6* overexpression (h*KLF6*^OE^) mice^9^ without injury, and 24 hours after one AAI injection. The single dose caused proximal tubule dilation, loss of brush border, and dedifferentiation, without cell death or functional changes (Supplemental Figure 1). Unsupervised clustering generated 23 clusters, which were identified using established cell type markers^14,15^ (Figure 1, A and B). Two clusters, InjuredPT-A and InjuredPT-B, were found almost exclusively in mice treated with AAI (Figure 1C) and coinciding with decreased numbers of PT-S3 cells (Figure 1D). Consistent with histologic analysis, few infiltrating/inflammatory cells were detected 24 hours post–AAI treatment (Figure 1D).

We first analyzed the effects of h*KLF6* overexpression without injury in normal proximal tubule clusters. Pathway analysis using genes upregulated in h*KLF6*^OE^ versus control mice demonstrated upregulated mitochondrial and ribosomal pathways and downregulated peroxisome proliferator-activated receptor signaling and mitochondrial long chain fatty acid *β*-oxidation in the PT-S3-2 cluster (Supplemental Figure 2, A and B). This may indicate that PT-S3-2 cells were more susceptible to injury in h*KLF6*^OE^ mice versus control mice. Gene ontology (GO) term analysis of genes upregulated in h*KLF6*^OE^ versus control also highlighted mitochondrial respiratory and metabolic processes (Supplemental Figure 2C).

**Figure 2:**
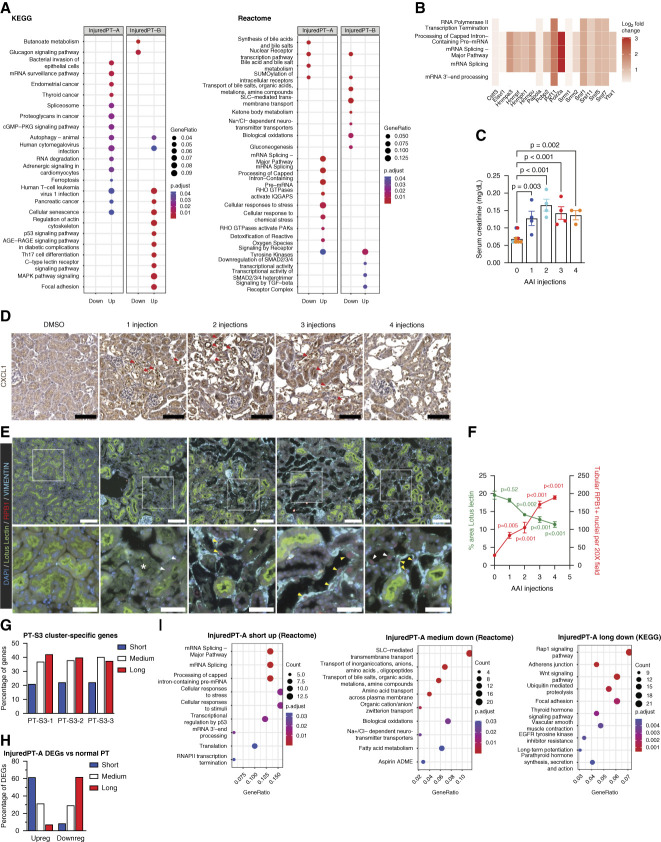
**Pathway analysis and cell fate of injured proximal tubule cells.** (A) Pathway analysis using DEGs specific to InjuredPT-A and InjuredPT-B clusters versus other proximal tubule clusters. GeneRatio=fraction of DEGs found in each gene set; p.adjust=false discovery rate. (B) DEGs contributing to upregulated Reactome pathways in InjuredPT-A cells. (C–E) Mice were given DMSO (vehicle) or a series of 1–4 AAI injections 3 days apart, with perfusion and collection of kidneys 72 hours after the last injection. (C) Serum creatinine for AAI versus DMSO (0 AAI injections). The zero AAI injections data points are amalgamated from one to four DMSO injections, colored to match the AAI doses. (D) Immunohistochemistry for CXCL1. Nuclei are counterstained with hematoxylin; scale bars=100 *µ*m. Red arrowheads: positivity for CXCL1. (E) Immunofluorescent staining for RPB1 and vimentin, with counterstaining for LL and DAPI in the time course of serial AAI injections. Scale bars=100 *µ*m (top) and 50 *µ*m (bottom). Asterisks: dying proximal tubule; white arrowheads: dedifferentiating proximal tubule (loss of LL brush border staining); yellow arrowheads: dedifferentiated proximal tubule (vimentin-positive). (F) Quantification of percentage area staining for LL and number of RPB1-positive tubular nuclei per 20× field in the time course of AAI injections. (C and F) One-way ANOVA with Dunnett multiple comparisons test. (G) Gene length categories of cluster-specific genes in normal PT-S3 clusters. (H) Length categories of differentially expressed genes in InjuredPT-A. (I) Pathway analyses of the upregulated short genes and downregulated medium and long genes in InjuredPT-A. AGE-RAGE, advanced glycan end products-receptor for advanced glycan end products; DAPI, 4′,6-diamidino-2-phenylindole; DEG, differentially expressed gene; EGFR, epidermal growth factor receptor; IQGAP, IQ motif containing GTPase-activating protein; KEGG, Kyoto Encyclopedia of Genes and Genomes; LL, lotus lectin; MAPK, mitogen-activated protein kinase; RHO, RAS homolog; RNAPII, RNA polymerase II; RPB1, RNA polymerase subunit B1; SLC, solute carrier; SUMO, small ubiquitin-like modifier.

Cross-matching with a published AKI snRNA-seq dataset showed some similarities between InjuredPT-B and Injured S1/S2/S3 and between InjuredPT-A and Severe Injured or maladaptive proximal tubule post–ischemia-reperfusion injury^14,16^ (Figure 1E and Supplemental Figure 3A). However, owing to the different injury models and timings used, overlap was limited. Confirming previous studies, mouse (m)*Klf6* was upregulated most highly in both the InjuredPT-A and InjuredPT-B clusters (Figure 1F and Supplemental Figure 3B). Pathways and GO terms enriched in normal proximal tubule were mostly absent in the injured proximal tubule clusters. Instead, InjuredPT-A was characterized by mRNA processing and splicing and translation, and InjuredPT-B by p53 signaling, cellular senescence, and cell cycle–related pathways (Supplemental Figure 4). Analysis of genes upregulated and downregulated in InjuredPT-A and InjuredPT-B versus normal proximal tubule clusters confirmed that normal proximal tubule metabolic and transport pathways were downregulated, while mRNA processing, splicing, and translation, and p53, Mapk signaling, and inflammatory pathways were upregulated in InjuredPT-A and InjuredPT-B, respectively (Figure 2A). In InjuredPT-A cells, upregulated genes in the mRNA-related pathways included heterogeneous nuclear ribonucleoproteins (Hnrnp's), splicing-related factors (Srsf's), and *Polr2a*, which encodes RNA polymerase subunit B1, the largest subunit of RNA polymerase II (RNAPII; Figure 2B). Significantly upregulated and downregulated genes in PT-S1-S2 and PT-S3 clusters from AAI-treated mice versus uninjured also showed upregulated p53 signaling and cell cycle–related and downregulated metabolic pathways, similarly to the InjuredPT-B cluster (Supplemental Figure 5A). There were small changes in cell cycle phase in InjuredPT-A and InjuredPT-B cells, with increases in S and G2/M phases (Supplemental Figure 5, B and C). To validate our findings, we reanalyzed data from an independent DNA damage–induced AKI scRNA-seq dataset, using cisplatin for 48 hours^17^ (Supplemental Figure 6). Subclustering of the proximal tubule clusters revealed injured clusters highly expressing *Krt20*, one of which also showed high *Klf6* and *Polr2a* expression. Pathway and GO analyses for this cluster versus the other proximal tubule clusters showed downregulation of metabolic processes and upregulation of mRNA processing pathways, similar to the InjuredPT-A cluster after AAI.

### InjuredPT-A and InjuredPT-B Clusters Had Different Fates

To determine the fates and potential contributions of the two injured proximal tubule clusters to fibrosis, we undertook immunofluorescence/immunohistochemical analyses for the top markers in InjuredPT-A (RPB1) and InjuredPT-B (CXCL1) across a time course of 1–4 AAI injections (Figure 2C). CXCL1 was associated with dying proximal tubule cells after one, two, and three injections, which were mostly cleared after four injections (Figure 2D). By contrast, RPB1 (*Polr2a*) was only minimally increased after one injection, and cells with high RPB1 expression had lower lotus lectin (LL) staining indicating early dedifferentiation, whereas tubules undergoing cell death did not have increased RBP1 expression, confirming that CXCL1 and RPB1 were expressed in different cells (Figure 2E and Supplemental Figure 7A). Over time, RPB1 expression became predominantly associated with injured dedifferentiated tubular cells either without LL staining or costaining with injury marker cytokeratin-20 (KRT-20; Supplemental Figure 7B) and vimentin (Figure 2E), suggesting that sustained high RPB1 expression in injured proximal tubules may be associated with dedifferentiation and/or failure of redifferentiation after injury. Fate tracing confirmed that tubules with high RPB1 lacking brush border were dedifferentiated proximal tubules (Supplemental Figure 7C). During the time course, RPB1-positive nuclei increased, whereas LL staining decreased, indicating that proximal tubule cells with RPB1-positive nuclei were preferentially retained relative to proximal tubule loss (Figure 2F). We also confirmed high proximal tubule RPB1 expression in two fibrotic models: RLDC and UUO (Supplemental Figure 8). Previous studies have shown that persistent high RPB1 expression and RNAPII stalling at DNA damage sites may cause a transcriptional switch, whereby short genes (<30 kb genomic span) are preferentially upregulated, whereas medium (>30 to <100 kb) and long (>100 kb) genes are suppressed.^18^ We therefore analyzed the gene lengths of normally expressed proximal tubule genes and those upregulated and downregulated in InjuredPT-A. In normal PT-S3 clusters, cluster-specific genes were approximately 20% short, approximately 35%–40% medium, and approximately 40%–45% long genes (Figure 2G). By contrast, of the upregulated genes in InjuredPT-A, approximately 60% were short, whereas in the downregulated genes, approximately 85% were either medium or long (Figure 2H). Pathway analysis using only short genes upregulated in InjuredPT-A revealed similar pathways to those using all upregulated genes (Figure 2I), whereas medium/long downregulated genes were involved in pathways related to normal proximal tubule cell function, including transport, metabolism, and cellular adhesion (Figure 2I). Thus, InjuredPT-A dedifferentiation may be mechanistically linked to increased RPB1 expression *via* a global transcriptional switch, which upregulates short genes and downregulates medium/long genes.

### Elevated *POLR2A* Expression Mediated Inflammation, Dedifferentiation, and Cell Cycle Arrest

**Figure 3:**
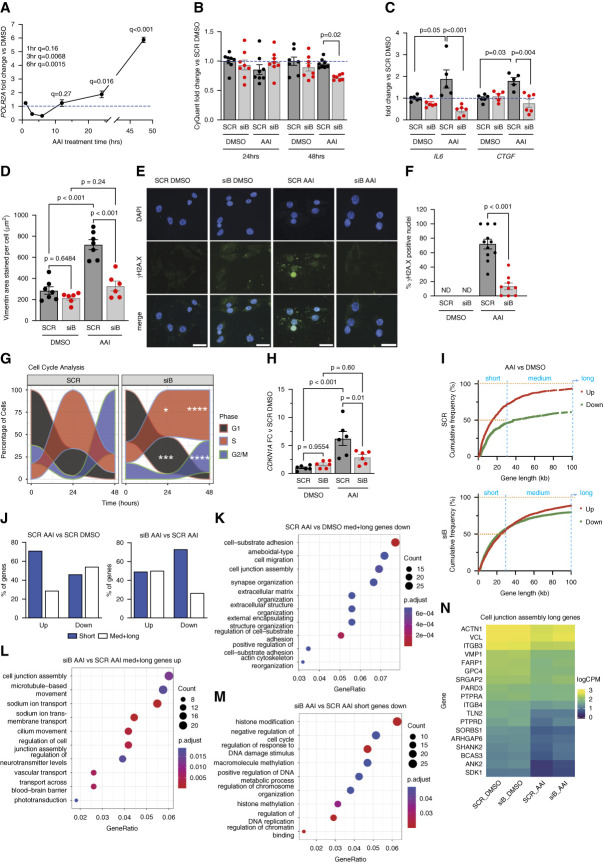
***POLR2A* knockdown decreases inflammation, dedifferentiation, DNA damage, and cell cycle arrest in injured kidney cells.** (A) Time course of *POLR2A* expression in HK-2 cells after AAI injury. FDR for unpaired *t* test at each time point, with two-stage step-up correction for multiple testing. (B) Quantification of HK-2 cell numbers with (siB) or without (SCR) *POLR2A* knockdown, treated with DMSO (vehicle) or AAI. One-way ANOVA with Sidak correction for multiple testing. (C) mRNA expression of *IL6* and *CTGF* in SCR and siB HK-2 cells treated with DMSO or AAI for 48 hours. One-way ANOVA with Sidak correction for multiple testing. (D) Quantification of area of vimentin stained per cell. One-way ANOVA with Sidak correction for multiple testing; *n*=6–7 fields per group from two independent experiments. (E) Immunofluorescence for *γ*H2A.X in SCR and siB HK-2 cells treated with DMSO or AAI for 48 hours. Nuclei are counterstained with DAPI; scale bars=50 *µ*m. (F) Quantification of percentage of *γ*H2A.X-positive nuclei per field, encompassing a total of 98–99 nuclei from two independent experiments. Student unpaired *t* test. (G and H) Cell cycle analysis (*n*=3 per group; G) and *CDKN1A* mRNA expression at 48 hours (H) in SCR and siB HK-2 cells treated with DMSO or AAI. siB versus SCR at each time point; one-way ANOVA with Sidak correction for multiple testing. (I) Cumulative frequency for significantly upregulated and downregulated genes for AAI versus DMSO treatment in SCR cells (top) and knockdown cells (bottom), sorted by gene length. (J) Percentages of upregulated and downregulated genes classified as short or medium/long in SCR cells treated with AAI versus DMSO (left), or knockdown versus SCR injured cells (right). (K–M) GO biologic process terms for medium/long genes downregulated in SCR cells treated with AAI versus DMSO (K), medium/long genes upregulated in knockdown versus SCR injured cells (L), and short genes downregulated in knockdown versus SCR injured cells (M). (N) Heatmap of genes from the “cell junction assembly” GO term. CPM, counts per million; CTGF, connective tissue growth factor; FC, fold change; FDR, false discovery rate; GO, gene ontology; HK-2, human kidney 2; SCR, scrambled.

*In vitro, POLR2A* mRNA expression in injured HK-2 cells was significantly downregulated at 3 hours and 6 hours of injury, but after 12 hours of injury, expression rapidly increased, sustained through 48 hours of injury (Figure 3A). *POLR2A* transient knockdown using short interfering RNA-B (Supplemental Figure 9A) resulted in increased cell death in AAI-treated cells (Figure 3B) versus control (scrambled [SCR]) AAI-treated cells after 48 hours. However, knockdown cells expressed lower levels of inflammatory cytokine *IL6*, and fibrotic marker *CTGF*, versus SCR cells post–AAI treatment (Figure 3C), indicating that knockdown of *POLR2A* may facilitate beneficial clearance of injured cells, and high expression of RPB1 may contribute to injury. Injured SCR cells had significantly increased vimentin expression, demonstrating increased dedifferentiation that was attenuated in knockdown cells (Figure 3D), suggesting that high RPB1 may drive dedifferentiation. Many SCR cells had DNA damage (*γ*H2A.X staining), whereas significantly fewer knockdown cells were *γ*H2A.X-positive, suggesting that cells with DNA damage were preferentially cleared after *POLR2A* knockdown (Figure 3, E and F). Cell cycle analysis showed that after 24 hours, injured SCR and knockdown cells predominantly shifted to S phase, although more knockdown cells were still in G1 versus control cells (Figure 3G and Supplemental Figure 9B). After 48 hours of injury, as expected, most SCR cells were in G2/M phase, consistent with the *γ*H2A.X expression, whereas most knockdown cells were still in S phase. Consistent with this, mRNA expression of *CDKN1A* (encoding p21) was significantly increased in injured SCR cells but significantly attenuated with *POLR2A* knockdown (Figure 3H). Thus, remaining knockdown cells had a more normal cell cycle distribution compared with SCR cells. To elucidate the cellular mechanisms, we undertook bulk RNA-seq at 24 hours, before the onset of cell death. Analysis of gene lengths of upregulated and downregulated genes in injured cells demonstrated that while most genes upregulated and downregulated in SCR cells were short and medium/long genes, respectively, gene length distributions of upregulated and downregulated genes in knockdown cells were similar (Figure 3I). Thus, the switch toward preferential transcription of short genes was partially reversed by knockdown of *POLR2A* (Figure 3J). Medium/long downregulated genes in SCR cells had enrichment of GO terms related to cellular adhesion, junctions, and structural organization, confirming this as a likely mechanism of the dedifferentiation in injured cells with high *POLR2A* expression (Figure 3K). Long genes upregulated in knockdown cells versus SCR cells were also enriched for cell junction, structural terms, and transport, indicating restoration of normal structure and functions in knockdown cells, whereas short genes downregulated in knockdown versus SCR cells constituted DNA damage, epigenetic, and cell cycle–related terms (Figure 3, L–N). Thus, dedifferentiation of injured cells *in vitro* was likely driven by the global transcriptional switch from long to short genes due to high *POLR2A* expression.

### KLF6 Contributed to Upregulation of *Polr2a*

**Figure 4:**
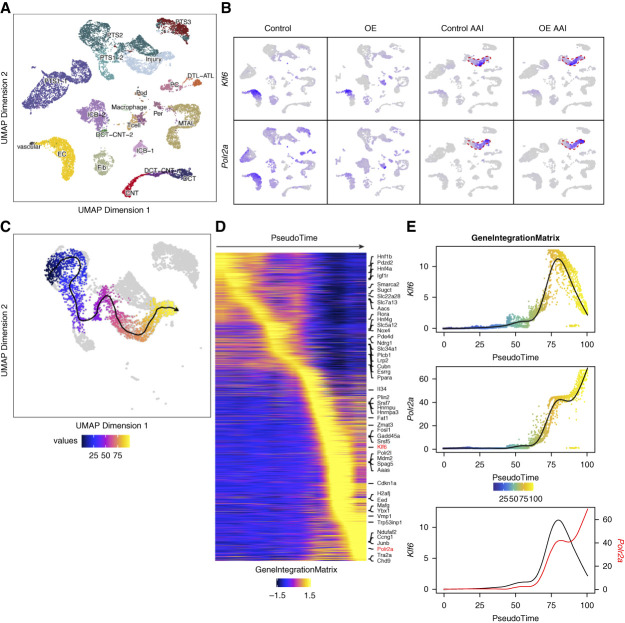
**Analysis of *Klf6* and *Polr2a* expression and dynamics.** (A) UMAP for integrated snRNA-seq and snATAC-seq of the same samples. (B) *Klf6* and *Polr2a* mRNA expression in the integrated UMAPs. Dashed red line denotes the injury cluster. (C) Pseudotime trajectory analysis from PTS2 to the injury cluster. (D) Gene integration matrix heatmap of the top 50 genes ranked by variance. (E) Expression of *Klf6* and *Polr2a* across pseudotime. PPAR, peroxisome proliferator-activated receptor; PTS2, proximal tubule segment 2; snATAC-seq, single nucleus assay for transposase-accessible chromatin sequencing.

To determine mechanisms of *Polr2a* upregulation in injured proximal tubule cells, we undertook snATAC-seq (Supplemental Figure 10, A–D). Integration with snRNA-seq data resulted in 21 clusters, including four proximal tubule clusters and one injured proximal tubule cluster with high *Klf6* and *Polr2a* expression (Figure 4, A and B, and Supplemental Figure 10, E–H). Pseudotime trajectory analysis from proximal tubule segment 2 to injury indicated that *Klf6* was upregulated earlier than *Polr2a*, consistent with potential regulation of *Polr2a* by KLF6 (Figure 4, C–E, and Supplemental Figure 11, A–C). In addition to open chromatin around the *Polr2a* promoter in all proximal tubule clusters containing a KLF6-binding site (Supplemental Figure 11D), peak-to-gene analysis showed that a strongly associated peak in intron 1 of *Polr2a* became newly accessible in the injury cluster, which may drive injury-related *Polr2a* upregulation (Supplemental Figure 11E). Exploration of transcription factor–binding sites in this novel region revealed multiple AP-1 sites and a KLF6-binding site (Supplemental Figure 11F). *Polr2a* expression was higher in the h*KLF6*^OE^ injury cluster versus control (Supplemental Figure 11G), and HK-2 cells with *KLF6* knockdown had significantly attenuated induction of *POLR2A* after 48 hours of injury, demonstrating a partial role for KLF6 in *POLR2A* induction after injury (Supplemental Figure 11H).

### h*KLF6*^TOE^ Mice Had Exacerbated Kidney Injury and Fibrosis after Nephrotoxic AKI

We sought to determine the effects of long-term tubular h*KLF6* overexpression on AAI-induced injury and *POLR2A*/RPB1 expression. Sustained tubule-specific h*KLF6* overexpression (h*KLF6*^TOE^) alone did not cause injury or loss of proximal tubules (Supplemental Figure 12, A–E). h*KLF6* was induced in the tubules, but not glomeruli, of h*KLF6*^TOE^ mice, and induction of h*KLF6* did not alter m*Klf6* expression (Supplemental Figure 12, F–H).

**Figure 5:**
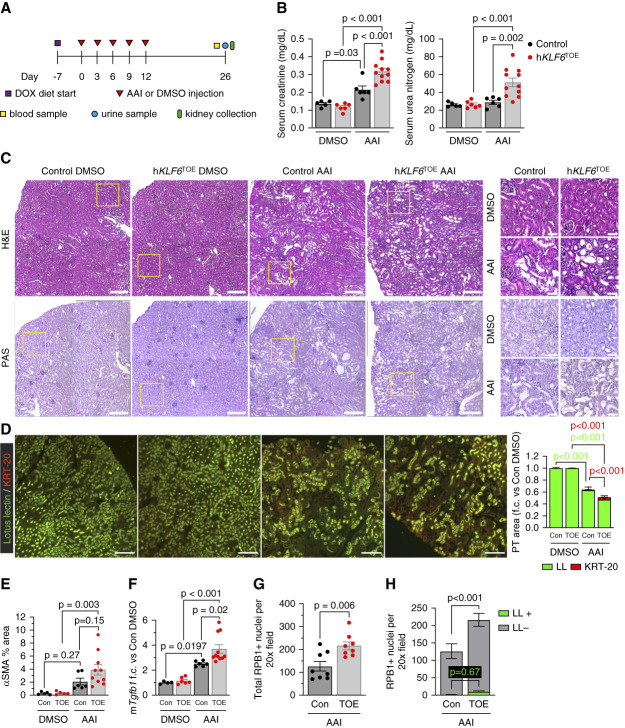
**Tubular h*KLF6* overexpression exacerbates nephrotoxic kidney injury and increases RPB1 expression.** (A) Protocol for induction of kidney injury using AAI. (B) Serum creatinine and urea nitrogen concentrations. (C) Histologic analysis using H&E and PAS stains. Scale bars=250 *µ*m (main images) and 50 *µ*m (insets). (D) Examination of proximal tubules using LL with immunofluorescence for KRT-20, and quantification of area staining positive; *n*=3–6 per group. Scale bars=250 *µ*m. (E) Quantification of *α*SMA area staining positive. (F) Kidney cortex mRNA expression of *Tgfb1*. (G) Quantification of total number of RPB1-positive (RPB1+) cells per 20× field in control (Con) and h*KLF6*^TOE^ (TOE) mice treated with AAI. (H) Quantification of RPB1-positive nuclei in LL positive (differentiated proximal tubules) and LL negative (dedifferentiated proximal tubules); *n*=8 per group. (B, D, E, and F) One-way ANOVA with Sidak correction for multiple testing; (G and H) Student unpaired *t* test. *α*SMA, alpha smooth muscle actin; DOX, doxycycline; H&E, hematoxylin and eosin; KRT-20, cytokeratin-20; PAS, periodic acid–Schiff; TOE, tubular overexpression.

To determine injury responses, we used a mild AAI protocol as our hypothesis was that h*KLF6*^TOE^ mice would have an exacerbated response to injury (Figure 5A). h*KLF6*^TOE^ mice had elevated serum creatinine and urea nitrogen concentrations and more extensive proximal tubule loss, inflammation, and protein casts compared with control mice (Figure 5, B and C). LL staining with immunofluorescence for KRT-20 showed more extensive loss of fully differentiated proximal tubules and more injured proximal tubules in h*KLF6*^TOE^ mice versus controls (Figure 5D). h*KLF6*^TOE^ mice also had increased alpha smooth muscle actin deposition and increased *Tgfb1* expression versus control mice after injury (Figure 5, E and F). Both m*Klf6* expression^9^ and h*KLF6* expression were significantly increased after injury, together resulting in a combined mouse and human (*Klf6*+*KLF6*) mRNA expression increase of approximately 75% in h*KLF6*^TOE^ compared with control mice after injury, which positively correlated with serum creatinine and urea nitrogen (Supplemental Figure 13, A–D). Consistent with our previous report demonstrating the role of KLF6 in regulating branched-chain amino acid (BCAA) catabolism,^9^ genes encoding BCAA catabolic enzymes pathway were more strongly downregulated in h*KLF6*^TOE^ versus control mice after injury (Supplemental Figure 13E).

To determine the influence of sustained h*KLF6* overexpression on RPB1 expression, we quantified the number of RPB1-positive nuclei in AAI-treated control and h*KLF6*^TOE^ mice (Supplemental Figure 14). There were significantly more RPB1-positive nuclei in h*KLF6*^TOE^ versus control mice (Figure 5G). Most RPB1-positive nuclei were located in LL-negative (*i.e*., dedifferentiated) proximal tubules, and the number of LL-negative/RPB1-positive nuclei was significantly higher in h*KLF6*^TOE^ mice versus control mice (Figure 5H). Together, these results suggest that sustained induction of tubule-specific KLF6 may drive high RPB1 expression, trapping proximal tubule cells in a dedifferentiated state.

### RPB1 Expression Was Upregulated in Human AKI and CKD

**Figure 6:**
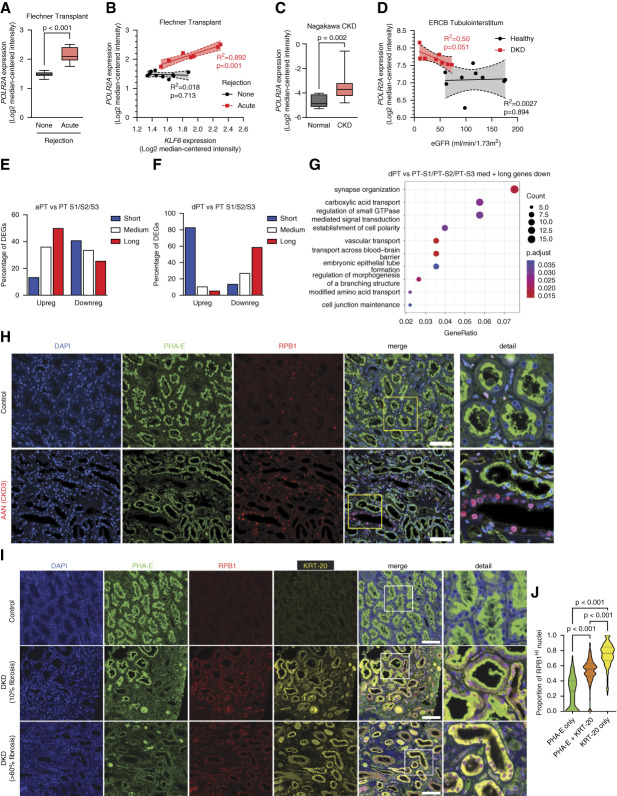
***POLR2A*/RPB1 expression in human AKI and CKD.** (A) Analysis of *POLR2A* expression levels from previously reported microarray data^19^ in post-transplant kidneys with (*n*=7) and without (*n*=10) acute rejection. One-tailed Mann–Whitney *U* test. (B) Correlation between *KLF6* and *POLR2A* expression in samples with and without acute rejection. Simple linear regression with 95% CI and with one-tailed *P* value. (C) Analysis of *POLR2A* in previously reported microarray data^20^ in healthy individuals (*n*=5) and patients with CKD (*n*=48). One-tailed Mann–Whitney *U* test. (D) Correlation between eGFR and *POLR2A* in tubulointerstitial samples from healthy individuals (*n*=9) and participants with DKD (*n*=8; ERCB, *via* Nephroseq). Simple linear regression with 95% CI and one-tailed *P* value. (E and F) Gene length categories of upregulated and downregulated genes in KPMP snRNA-seq dataset aPT (E) and dPT (F) versus PT-S1/PT-S2/PT-S3 clusters. (G) GO biologic process terms for medium/long genes downregulated in dPT. (H) Immunofluorescent staining for RPB1 with counterstaining for PHA-E and DAPI in a control participant and a patient with CKD and AA exposure. Scale bars=100 *µ*m. (I) Coimmunofluorescent staining for RPB1 and KRT-20 with counterstaining for PHA-E and DAPI in a control participant and patients with DKD. Scale bars=100 *µ*m. (J) Quantification of proportion of RPB1^HI^ nuclei in PHA-E–positive, KRT-20–positive, and double-positive tubules from five control and 14 DKD patients. One-way ANOVA with Tukey multiple comparisons test. aPT, adaptive proximal tubule; CI, confidence interval; DKD, diabetic kidney disease; dPT, degenerative proximal tubule; ERCB, European Renal cDNA Bank; KPMP, Kidney Precision Medicine Project; PHA-E, *Phaseolus vulgaris* Erythroagglutinin.

To determine correlations between *POLR2A*/RPB1 expression and AKI and CKD in human patients, we first undertook analysis of microarray data deposited in the Nephroseq database. Analysis of a transplant dataset^19^ showed significantly higher *POLR2A* expression in acute rejection versus no rejection samples (Figure 6A). Furthermore, *POLR2A* expression significantly positively correlated with *KLF6* expression in acute rejection samples, but not in no rejection samples (Figure 6B). *POLR2A* expression was also significantly higher in CKD patient kidneys versus normal kidneys^20^ (Figure 6C), and tubulointerstitial *POLR2A* expression was negatively correlated with eGFR in kidney biopsies in patients with DKD, but not in healthy participants (Figure 6D). We then undertook analysis of gene lengths in the adaptive proximal tubule (aPT) and degenerative proximal tubule (dPT) clusters in snRNA-seq samples from Kidney Precision Medicine Project. Both the aPT and dPT had increased expression of *KLF6* compared with normal proximal tubule clusters (PT-S1, PT-S2, and PT-S3 combined). However, whereas the aPT cells did not show any shift in transcription toward short genes, the dPT cells showed the same signature of gene length alterations as our InjuredPT-A, with most upregulated genes being short genes (approximately 83%) and most downregulated genes being medium or long (approximately 86%), suggesting that this transcriptional switch is indeed associated with lack of an adaptive response (Figure 6, E and F). Downregulated medium and long genes in dPT were enriched for differentiation, polarity, and cell junction–related GO terms (Figure 6G). We undertook immunofluorescent staining in normal samples and patients with CKD confirmed or suspected to have been exposed to AA.^10,11^ In fully differentiated *Phaseolus vulgaris* Erythroagglutinin (PHA-E)–positive tubules, RPB1 expression was rarely detected above the background tubular autofluorescence in both control and CKD samples (Figure 6H and Supplemental Figure 15). In patients with CKD, many PHA-E–negative tubules had high RPB1 expression. We also undertook immunofluorescence for RPB1 and KRT-20 in control and samples from DKD patients with varying degrees of fibrosis and kidney function. Control samples contained predominantly PHA-E–positive, KRT-20–negative tubules, whereas DKD samples contained predominantly KRT-20–positive or double-positive (PHA-E and KRT-20) tubules (Figure 6I and Supplemental Figure 16, A and B). Quantification of the proportion of RPB1^HI^ nuclei per tubule showed that KRT-20–positive and double-positive tubules had higher proportions of RPB1^HI^ nuclei than PHA-E–positive tubules (Figure 6J and Supplemental Figure 16C).

## Discussion

To determine early pathways driven by KLF6 in AKI, we undertook snRNA-seq of kidneys from control and h*KLF6*^OE^ mice at 24 hours after one dose of AAI, with exclusion parameters of <200 or >6000 genes, or >10% mitochondrial genes.^21,22^ We identified two injured proximal tubule clusters with high m*Klf6* expression (Figure 1F). InjuredPT-A cells had enrichment for mRNA processing and splicing, and translation pathways, while InjuredPT-B cells had enrichment for p53 signaling and inflammatory and fibrotic cytokines (Figure 2A). These injured proximal tubule clusters were confirmed to be different by immunofluorescence and immunohistochemistry staining, with different cell fates (Figure 2, D and E). This suggests cell context-dependent roles and/or target genes for KLF6-mediated transcriptional activation or repression, which may depend on coexpression and/or interaction of other transcription factors, or differential chromatin accessibility.

The InjuredPT-B cluster was characterized by p53 signaling, and cells in this cluster appeared to undergo cell death in a time course of AAI (Figure 2D). KLF6 interacts with p53 through protein–protein interactions to regulate expression of downstream genes in colorectal cancer cells.^23^ However, different studies have reported opposite roles of p53 in the pathogenesis of AAI nephropathy.^24,25^ Since InjuredPT-B cells underwent cell death, it is unlikely that this cluster contributed to the phenotypic difference between control and h*KLF6*^TOE^ mice in post–AAI fibrosis (Figure 5).

The InjuredPT-A cluster was characterized by mRNA processing and splicing, and the highest upregulated gene in the cluster was *Polr2a*, encoding RPB1, the largest subunit of RNAPII, which is necessary for transcription of all protein-encoding genes and noncoding RNA genes. We show here that RPB1 upregulation likely plays a pathogenic role in injured proximal tubule cells. Knockdown of *POLR2A* in HK-2 cells led to lower expression of inflammatory and fibrotic cytokines (*IL6* and *CTGF*), less cellular dedifferentiation, and less G2/M cell cycle arrest versus injured cells with high RPB1 expression (Figure 3). This was likely due to increased clearance of cells with DNA damage, as shown by increased cell death but less DNA damage in the remaining cells after *POLR2A* knockdown (Figure 3F). Changes in cell cycle in response to changes in *POLR2A* expression have been shown in cancer cells, with *POLR2A* knockdown and overexpression respectively decreasing and increasing the proportion of cells in G2/M phase.^26^ Correlations between dedifferentiated proximal tubule cells and high RPB1 expression were confirmed in other mouse models of AKI and fibrosis and in samples from human CKD patients. However, our studies included only a small number of patients with DKD and did not encompass other etiologies of CKD, which should be included in additional studies, to determine whether this is a common mechanism in CKD. Future studies will be needed to elucidate the cellular mechanisms by which the sustained high expression of RPB1 causes injured proximal tubule cells to remain in a dedifferentiated state. Proximal tubule cells that acquire a maladaptive dedifferentiation state are drivers of fibrosis.^4,5^ Thus, clearance of such cells, as occurred with *POLR2A* knockdown *in vitro*, may have a beneficial effect, analogous to the theory behind the development of senolytic drugs. We also noted high expression in some interstitial cells, and further studies should address the role of RPB1 in these cells.

We observed an interesting dynamic response of *POLR2A* mRNA expression after AAI *in vitro* such that *POLR2A* mRNA was initially downregulated until 12 hours after injury and then subsequently was highly and rapidly induced. After ultraviolet-induced DNA damage in HEK293 cells, RPB1 protein must be rapidly degraded after DNA damage to allow effective DNA repair, but RPB1 expression must then be restored in order for global transcription to restart.^18,27^ However, RNAPII can also become stalled at DNA lesions. Further studies will be needed to determine whether high RPB1 expression in dedifferentiated proximal tubule cells is due to continued high transcription, RPB1 protein stabilization, or RNAPII stalling. Persistently stalled RNAPII after ultraviolet damage *in vitro* led to a transcriptional switch toward preferential transcription of short genes and downregulation of medium and long genes.^18^ We found a similar switch both *in vivo* in InjuredPT-A cells and *in vitro*, which was partially reversed by *POLR2A* knockdown. Furthermore, downregulated genes were strongly associated with structure and functions of differentiated proximal tubule cells. Thus, persistent stalling of RNAPII may drive proximal tubular dedifferentiation through a global transcriptional switch. However, further studies will be needed to confirm this mechanism. We also detected the same pattern of high RPB1 expression in dedifferentiated proximal tubule in kidneys after UUO, which do not have direct DNA damage, in addition to AAI and cisplatin, in which DNA lesions are formed. Further mechanistic studies will be needed to show whether RPB1 upregulation in UUO kidneys is induced by indirect DNA damage, *e.g*., from reactive oxygen species, or whether upregulation can also occur without DNA damage, as a more general phenomenon. Irrespective of direct or indirect DNA damage in AAI, cisplatin, and UUO experimental models, the common feature is upregulation of KLF6.

Sustained tubular overexpression of h*KLF6* in mice exacerbated injury and fibrosis after AAI, associated with increased RPB1 expression and reduction of fully differentiated proximal tubules (Figure 5). Furthermore, HK-2 cells with *KLF6* knockdown had less upregulation of *POLR2A* in response to AKI treatment. Mapping of KLF6-binding sites from published chromatin immunoprecipitation sequencing data showed KLF6 binding at the *Polr2a* promoter, and our integrated snRNA-seq/snATAC-seq data revealed an additional region in intron 1 of *Polr2a* that became open in injured proximal tubule cells, also containing a KLF6-binding site. Transcriptional mechanisms controlling the total level of RPB1 and RNAPII both in normal cells and in response to DNA damage are not clearly understood.^28^ Our data together are strongly suggestive of a role for KLF6 in transcriptional upregulation of *Polr2a* mRNA in injured proximal tubule cells, where KLF6 is itself also upregulated. However, this would require further validation of chromatin opening and KLF6 binding, *e.g*., using chromatin immunoprecipitation–quantitative PCR. We also only used one method to induce AKI and fibrosis in the h*KLF6*^TOE^ mice, and it will be important to determine whether this mechanism occurs in other models of direct and indirect DNA damage. More studies will be needed to understand whether other cofactors such as activating protein-1 family members are necessary for the rapid and high transcriptional upregulation of *Polr2a* in injured proximal tubule cells in early AKI.

In conclusion, our studies show that *Polr2a*/RPB1 is highly upregulated in injured proximal tubule cells and that sustained high expression of RPB1 appears to promote a dedifferentiated proximal tubule phenotype. KLF6 likely plays a role in this maladaptive upregulation of RPB1, and overexpression of KLF6 in mice led to significantly increased RPB1 and increased kidney injury and fibrosis.

## Supplementary Material

SUPPLEMENTARY MATERIAL

## Data Availability

Previously published data were used for this study. Original data created for the study are or will be available in a persistent repository upon publication. Chen, Z, Li, Y, Yuan, Y, Lai, K, Ye, K, Lin, Y, Lan, R, Chen, H, Xu, Y: Single-cell sequencing reveals homogeneity and heterogeneity of the cytopathologic mechanisms in different etiology-induced AKI. *Cell Death Dis,* 14: 318, 2023. Experimental Data. Gene Expression Omnibus; GitHub. snRNA-seq, single nucleus assay for transposase-accessible chromatin sequencing, and bulk RNA-seq data were deposited in the National Center for Biotechnology Information Gene Expression Omnibus database under accession numbers GSE268344 (snRNA-seq), GSE268605 (snATAC-seq), and GSE282647 (bulk RNA-seq). R code is available from: https://github.com/MallipattuLab/MallipattuLab-KLF6-POLR2A-manuscript.

## Supplemental Material

Supplemental Methods

Supplemental Figure 1. Phenotype and quality control metrics in snRNA-seq samples.

Supplemental Figure 2. Pathway and GO term analysis of DEGs in h*KLF6*^OE^ versus control mice for proximal tubule clusters.

Supplemental Figure 3. Expression of marker genes.

Supplemental Figure 4. Pathway and GO term analysis of cluster-specific DEGs in normal and injured proximal tubule clusters.

Supplemental Figure 5. KEGG pathway and cell cycle analyses.

Supplemental Figure 6. Identification of a Klf6/Polr2a injured proximal tubule cluster in a cisplatin scRNA-seq dataset.

Supplemental Figure 7. RPB1 expression in AAI, RLDC, and UUO models.

Supplemental Figure 8. RPB1 expression in UUO and RLDC models.

Supplemental Figure 9. Knockdown of POLR2A in HK-2 cells.

Supplemental Figure 10. Quality control metrics and cluster assignment in snATAC-seq samples.

Supplemental Figure 11. Potential role for KLF6 in regulation of POLR2A expression.

Supplemental Figure 12. Tubular hKLF6 overexpression alone does not cause kidney injury.

Supplemental Figure 13. Klf6 expression, kidney function, and BCAA catabolism gene expression in control and hKLF6TOE mice treated with AAI.

Supplemental Figure 14. RPB1 immunofluorescence in control and hKLF6^TOE^ mice.

Supplemental Figure 15. RPB1 expression in patients with CKD.

Supplemental Figure 16. RPB1 expression in control and DKD patients.

snRNA-seq cluster-specific DEGs for each cluster
