# Supplementary material for: Krüppel-Like Factor 6 Induces RNA Polymerase II Subunit RPB1 to Promote Kidney Injury
Source: J Am Soc Nephrol. 2025 May 6;36(10):1914–27. doi: 10.1681/ASN.0000000722 (PMC12499623; doi:10.1681/ASN.0000000722)
Supplement: SUPPLEMENTARY MATERIAL [file jasn-36-1914-s002.pdf]

ASN
American Society of Nephrology
JASN
Journal of the American Society of Nephrology
KIDNEY360
Accessing Our World From Every Angle
CJASN
Clinical Journal of the American Society of Nephrology

ASN Journal Disclosure Form

As per ASN journal policy, I have disclosed any financial relationships or commitments I have held in the past 36 months as included below. I have listed my Current Employer below to indicate there is a relationship requiring disclosure. If no relationship exists, my Current Employer is not listed.

T. Bahadur reports the following:
Employer: Stony Brook Medicine - Division of Nephrology

I understand that the information above will be published within the journal article, if accepted, and that failure to comply and/or to accurately and completely report the potential financial conflicts of interest could lead to the following: 1) Prior to publication, article rejection, or 2) Post-publication, sanctions ranging from, but not limited to, issuing a correction, reporting the inaccurate information to the authors' institution, banning authors from submitting work to ASN journals for varying lengths of time, and/or retraction of the published work.

Name: Tej Bahadur
Manuscript ID: JASN-2024-001533R2
Manuscript Title: Krüppel-like Factor 6 Induces RNA Polymerase II Subunit RPB1 to Promote Kidney Injury
Date of Completion: April 19, 2025
Disclosure Updated Date: May 21, 2024

ASN American Society of Nephrology | JASN Journal of the American Society of Nephrology | KIDNEY360 Accessing Our World From Every Angle | CJASN Clinical Journal of the American Society of Nephrology

ASN Journal Disclosure Form

As per ASN journal policy, I have disclosed any financial relationships or commitments I have held in the past 36 months as included below. I have listed my Current Employer below to indicate there is a relationship requiring disclosure. If no relationship exists, my Current Employer is not listed.

R. Bronstein has nothing to disclose.

I understand that the information above will be published within the journal article, if accepted, and that failure to comply and/or to accurately and completely report the potential financial conflicts of interest could lead to the following: 1) Prior to publication, article rejection, or 2) Post-publication, sanctions ranging from, but not limited to, issuing a correction, reporting the inaccurate information to the authors' institution, banning authors from submitting work to ASN journals for varying lengths of time, and/or retraction of the published work.

Name: Robert Bronstein
Manuscript ID: JASN-2024-001533R1
Manuscript Title: Krüppel-like factor 6 induces RNA polymerase II subunit RPB1 to promote kidney injury
Date of Completion: March 17, 2025
Disclosure Updated Date: May 21, 2024

ASN American Society of Nephrology
JASN Journal of the American Society of Nephrology
KIDNEY360 Accessing Our World From Every Angle
CJASN Clinical Journal of the American Society of Nephrology

ASN Journal Disclosure Form

As per ASN journal policy, I have disclosed any financial relationships or commitments I have held in the past 36 months as included below. I have listed my Current Employer below to indicate there is a relationship requiring disclosure. If no relationship exists, my Current Employer is not listed.

M. Davis has nothing to disclose.

I understand that the information above will be published within the journal article, if accepted, and that failure to comply and/or to accurately and completely report the potential financial conflicts of interest could lead to the following: 1) Prior to publication, article rejection, or 2) Post-publication, sanctions ranging from, but not limited to, issuing a correction, reporting the inaccurate information to the authors' institution, banning authors from submitting work to ASN journals for varying lengths of time, and/or retraction of the published work.

Name: Merin Davis
Manuscript ID: JASN-2024-001533R1
Manuscript Title: Krüppel-like factor 6 induces RNA polymerase II subunit RPB1 to promote kidney injury
Date of Completion: March 25, 2025
Disclosure Updated Date: March 25, 2025

ASN American Society of Nephrology · JASN Journal of the American Society of Nephrology · KIDNEY360 Accessing Our World From Every Angle · CJASN Clinical Journal of the American Society of Nephrology

ASN Journal Disclosure Form

As per ASN journal policy, I have disclosed any financial relationships or commitments I have held in the past 36 months as included below. I have listed my Current Employer below to indicate there is a relationship requiring disclosure. If no relationship exists, my Current Employer is not listed.

S. DiMartino reports the following:
Employer: StonyBrook Medicine -Nephrology department

I understand that the information above will be published within the journal article, if accepted, and that failure to comply and/or to accurately and completely report the potential financial conflicts of interest could lead to the following: 1) Prior to publication, article rejection, or 2) Post-publication, sanctions ranging from, but not limited to, issuing a correction, reporting the inaccurate information to the authors' institution, banning authors from submitting work to ASN journals for varying lengths of time, and/or retraction of the published work.

Name: Samaneh DiMartino
Manuscript ID: JASN-2024-001533R1
Manuscript Title: Krüppel-like factor 6 induces RNA polymerase II subunit RPB1 to promote kidney injury
Date of Completion: March 17, 2025
Disclosure Updated Date: March 17, 2025

ASN American Society of Nephrology
JASN Journal of the American Society of Nephrology
KIDNEY360 Accessing Our World From Every Angle
CJASN Clinical Journal of the American Society of Nephrology

ASN Journal Disclosure Form

As per ASN journal policy, I have disclosed any financial relationships or commitments I have held in the past 36 months as included below. I have listed my Current Employer below to indicate there is a relationship requiring disclosure. If no relationship exists, my Current Employer is not listed.

N. Gujarati reports the following:
Employer: Stony Brook University

I understand that the information above will be published within the journal article, if accepted, and that failure to comply and/or to accurately and completely report the potential financial conflicts of interest could lead to the following: 1) Prior to publication, article rejection, or 2) Post-publication, sanctions ranging from, but not limited to, issuing a correction, reporting the inaccurate information to the authors' institution, banning authors from submitting work to ASN journals for varying lengths of time, and/or retraction of the published work.

Name: Nehaben A. Gujarati
Manuscript ID: JASN-2024-001533R1
Manuscript Title: "Krüppel-like factor 6 induces RNA polymerase II subunit RPB1 to promote kidney injury"
Date of Completion: March 17, 2025
Disclosure Updated Date: May 20, 2024

ASN
American Society of Nephrology
JASN
Journal of the American Society of Nephrology
KIDNEY360
Accessing Our World From Every Angle
CJASN
Clinical Journal of the American Society of Nephrology

ASN Journal Disclosure Form

As per ASN journal policy, I have disclosed any financial relationships or commitments I have held in the past 36 months as included below. I have listed my Current Employer below to indicate there is a relationship requiring disclosure. If no relationship exists, my Current Employer is not listed.

Y. Guo reports the following:
Employer: Stony Brook University

I understand that the information above will be published within the journal article, if accepted, and that failure to comply and/or to accurately and completely report the potential financial conflicts of interest could lead to the following: 1) Prior to publication, article rejection, or 2) Post-publication, sanctions ranging from, but not limited to, issuing a correction, reporting the inaccurate information to the authors' institution, banning authors from submitting work to ASN journals for varying lengths of time, and/or retraction of the published work.

Name: Yiqing Guo
Manuscript ID: JASN-2024-001533R1
Manuscript Title: Krüppel-like factor 6 induces RNA polymerase II subunit RPB1 to promote kidney injury,
Date of Completion: March 17, 2025
Disclosure Updated Date: May 20, 2024

ASN American Society of Nephrology
JASN Journal of the American Society of Nephrology
KIDNEY360 Accessing Our World From Every Angle
CJASN Clinical Journal of the American Society of Nephrology

ASN Journal Disclosure Form

As per ASN journal policy, I have disclosed any financial relationships or commitments I have held in the past 36 months as included below. I have listed my Current Employer below to indicate there is a relationship requiring disclosure. If no relationship exists, my Current Employer is not listed.

M. Hanubal has nothing to disclose.

I understand that the information above will be published within the journal article, if accepted, and that failure to comply and/or to accurately and completely report the potential financial conflicts of interest could lead to the following: 1) Prior to publication, article rejection, or 2) Post-publication, sanctions ranging from, but not limited to, issuing a correction, reporting the inaccurate information to the authors' institution, banning authors from submitting work to ASN journals for varying lengths of time, and/or retraction of the published work.

Name: Maanasa S. Hanubal
Manuscript ID: JASN-2024-001533R1
Manuscript Title: Krüppel-like Factor 6 Induces RNA Polymerase II Subunit RPB1 to Promote Kidney Injury
Date of Completion: April 2, 2025
Disclosure Updated Date: May 16, 2024

ASN Journal Disclosure Form

As per ASN journal policy, I have disclosed any financial relationships or commitments I have held in the past 36 months as included below. I have listed my Current Employer below to indicate there is a relationship requiring disclosure. If no relationship exists, my Current Employer is not listed.

S. Mallipattu reports the following:
Employer: Stony Brook Medicine; Consultancy: Wildwood Therapeutics, Inc.; L.E.K. Consulting; Dedham Group; Graticule;; Research Funding: Dialysis Clinic Inc.; Patents or Royalties: Krüppel-like factor 15 (KLF15) Small Molecule Agonists in Kidney Disease. US 63/018.247. 2023.; and Advisory or Leadership Role: Clinically Integrated Network, Board Member (Accountable Care Organization, LLC Stony Brook Medicine);.

I understand that the information above will be published within the journal article, if accepted, and that failure to comply and/or to accurately and completely report the potential financial conflicts of interest could lead to the following: 1) Prior to publication, article rejection, or 2) Post-publication, sanctions ranging from, but not limited to, issuing a correction, reporting the inaccurate information to the authors' institution, banning authors from submitting work to ASN journals for varying lengths of time, and/or retraction of the published work.

Name: Sandeep K. Mallipattu
Manuscript ID: JASN-2024-001533R1
Manuscript Title: Krüppel-like factor 6 induces RNA polymerase II subunit RPB1 to promote kidney injury
Date of Completion: March 18, 2025
Disclosure Updated Date: March 18, 2025

ASN American Society of Nephrology
JASN Journal of the American Society of Nephrology
KIDNEY360 Accessing Our World From Every Angle
CJASN Clinical Journal of the American Society of Nephrology

ASN Journal Disclosure Form

As per ASN journal policy, I have disclosed any financial relationships or commitments I have held in the past 36 months as included below. I have listed my Current Employer below to indicate there is a relationship requiring disclosure. If no relationship exists, my Current Employer is not listed.

B. Owusu Frimpong reports the following:
Employer: Yale University

I understand that the information above will be published within the journal article, if accepted, and that failure to comply and/or to accurately and completely report the potential financial conflicts of interest could lead to the following: 1) Prior to publication, article rejection, or 2) Post-publication, sanctions ranging from, but not limited to, issuing a correction, reporting the inaccurate information to the authors' institution, banning authors from submitting work to ASN journals for varying lengths of time, and/or retraction of the published work.

Name: Bismark Owusu Frimpong
Manuscript ID: JASN-2024-001533
Manuscript Title: Krüppel-like factor 6 induces RNA polymerase II subunit RPB1 to promote kidney injury
Date of Completion: January 17, 2025
Disclosure Updated Date: January 17, 2025

ASN
American Society of Nephrology
JASN
Journal of the American Society of Nephrology
KIDNEY360
Accessing Our World From Every Angle
CJASN
Clinical Journal of the American Society of Nephrology

ASN Journal Disclosure Form

As per ASN journal policy, I have disclosed any financial relationships or commitments I have held in the past 36 months as included below. I have listed my Current Employer below to indicate there is a relationship requiring disclosure. If no relationship exists, my Current Employer is not listed.

S. Piret reports the following:
Employer: Stony Brook University

I understand that the information above will be published within the journal article, if accepted, and that failure to comply and/or to accurately and completely report the potential financial conflicts of interest could lead to the following: 1) Prior to publication, article rejection, or 2) Post-publication, sanctions ranging from, but not limited to, issuing a correction, reporting the inaccurate information to the authors' institution, banning authors from submitting work to ASN journals for varying lengths of time, and/or retraction of the published work.

Name: Sian E. Piret
Manuscript ID: JASN-2024-001533R1
Manuscript Title: Krüppel-like factor 6 induces RNA polymerase II subunit RPB1 to promote kidney injury
Date of Completion: March 17, 2025
Disclosure Updated Date: March 17, 2025

ASN
American Society of Nephrology
JASN
Journal of the American Society of Nephrology
KIDNEY360
Accessing Our World From Every Angle
CJASN
Clinical Journal of the American Society of Nephrology

ASN Journal Disclosure Form

As per ASN journal policy, I have disclosed any financial relationships or commitments I have held in the past 36 months as included below. I have listed my Current Employer below to indicate there is a relationship requiring disclosure. If no relationship exists, my Current Employer is not listed.

A. Rath reports the following:
Employer: Stony Brook University

I understand that the information above will be published within the journal article, if accepted, and that failure to comply and/or to accurately and completely report the potential financial conflicts of interest could lead to the following: 1) Prior to publication, article rejection, or 2) Post-publication, sanctions ranging from, but not limited to, issuing a correction, reporting the inaccurate information to the authors' institution, banning authors from submitting work to ASN journals for varying lengths of time, and/or retraction of the published work.

Name: Asha Rath
Manuscript ID: JASN-2024-001533R1
Manuscript Title: Krüppel-like factor 6 induces RNA polymerase II subunit RPB1 to promote kidney injury
Date of Completion: March 17, 2025
Disclosure Updated Date: May 20, 2024

ASN American Society of Nephrology
JASN Journal of the American Society of Nephrology
KIDNEY360 Accessing Our World From Every Angle
CJASN Clinical Journal of the American Society of Nephrology

ASN Journal Disclosure Form

As per ASN journal policy, I have disclosed any financial relationships or commitments I have held in the past 36 months as included below. I have listed my Current Employer below to indicate there is a relationship requiring disclosure. If no relationship exists, my Current Employer is not listed.

M. Revelo Penafiel reports the following:
Patents or Royalties: Elsevier.

I understand that the information above will be published within the journal article, if accepted, and that failure to comply and/or to accurately and completely report the potential financial conflicts of interest could lead to the following: 1) Prior to publication, article rejection, or 2) Post-publication, sanctions ranging from, but not limited to, issuing a correction, reporting the inaccurate information to the authors' institution, banning authors from submitting work to ASN journals for varying lengths of time, and/or retraction of the published work.

Name: Monica Patricia Revelo Penafiel
Manuscript ID: JASN-2024-001533R1
Manuscript Title: Krüppel-like factor 6 induces RNA polymerase II subunit RPB1 to promote kidney injury,"
Date of Completion: March 17, 2025
Disclosure Updated Date: March 17, 2025

ASN American Society of Nephrology
JASN Journal of the American Society of Nephrology
KIDNEY360 Accessing Our World From Every Angle
CJASN Clinical Journal of the American Society of Nephrology

ASN Journal Disclosure Form

As per ASN journal policy, I have disclosed any financial relationships or commitments I have held in the past 36 months as included below. I have listed my Current Employer below to indicate there is a relationship requiring disclosure. If no relationship exists, my Current Employer is not listed.

J. Wang reports the following:
Employer: Stonybrook University

I understand that the information above will be published within the journal article, if accepted, and that failure to comply and/or to accurately and completely report the potential financial conflicts of interest could lead to the following: 1) Prior to publication, article rejection, or 2) Post-publication, sanctions ranging from, but not limited to, issuing a correction, reporting the inaccurate information to the authors' institution, banning authors from submitting work to ASN journals for varying lengths of time, and/or retraction of the published work.

Name: Jiakang Wang
Manuscript ID: JASN-2024-001533R2
Manuscript Title: Krüppel-like Factor 6 Induces RNA Polymerase II Subunit RPB1 to Promote Kidney Injury
Date of Completion: April 14, 2025
Disclosure Updated Date: May 13, 2024
