# Supplementary material for: Krüppel-Like Factor 6 Induces RNA Polymerase II Subunit RPB1 to Promote Kidney Injury
Source: J Am Soc Nephrol. 2025 May 6;36(10):1914–27. doi: 10.1681/ASN.0000000722 (PMC12499623; doi:10.1681/ASN.0000000722)

Krüppel-like factor 6 induces RNA polymerase II subunit RPB1 to promote kidney injury – Supplemental Material

Table of Contents

Supplemental Methods
Supplemental Figure 1. Phenotype and quality control metrics in snRNA-seq samples.
Supplemental Figure 2. Pathway and GO term analysis of DEGs in hKLF6OE versus control mice for PT clusters.
Supplemental Figure 3. Expression of marker genes.
Supplemental Figure 4. Pathway and GO term analysis of cluster-specific DEGs in normal and injured PT clusters.
Supplemental Figure 5. KEGG pathway and cell cycle analyses.
Supplemental Figure 6. Identification of a Klf6/Polr2a injured PT cluster in a cisplatin scRNA-seq dataset.
Supplemental Figure 7. RPB1 expression in AAI, repeated low-dose cisplatin (RLDC) and unilateral ureteral obstruction (UUO) models.
Supplemental Figure 8. RPB1 expression in unilateral ureteral obstruction (UUO) and repeated low-dose cisplatin (RLDC) models.
Supplemental Figure 9. Knockdown of POLR2A in HK-2 cells.
Supplemental Figure 10. Quality control metrics and cluster assignment in snATAC-seq samples.
Supplemental Figure 11. Potential role for KLF6 in regulation of POLR2A expression.
Supplemental Figure 12. Tubular hKLF6 overexpression alone does not cause kidney injury.
Supplemental Figure 13. Klf6 expression, kidney function, and branched-chain amino acid (BCAA) catabolism gene expression in control and hKLF6TOE mice treated with AAI.
Supplemental Figure 14. RPB1 immunofluorescence in control and hKLF6^{TOE} mice.
Supplemental Figure 15. RPB1 expression in CKD patients.
Supplemental Figure 16. RPB1 expression in control and diabetic kidney disease (DKD) patients.
snRNA-seq cluster-specific DEGs for each cluster.

SUPPLEMENTAL METHODS

Generation of tubule-specific human KLF6 overexpression (h*KLF6*^{TOE}) mice

Mice carrying the tetracycline-response element (TRE-h*KLF6*) transgene on an FVB/N background[1] were bred with mice carrying the reverse tetracycline transactivator (rtTA) transgene under the control of the Pax8 promoter (*Pax8*-rtTA) also on the FVB/N background (Jackson Labs). Genotyping by tail biopsy and PCR was performed prior to weaning as previously described[2]. Mice carrying only the TRE-h*KLF6* transgene were used as controls. All mice were fed diet containing 200mg/kg doxycycline (DOX) starting at age 6 weeks (aging study) or one week prior to aristolochic acid I (AAI) injections (fibrosis study), and remained on DOX continuously.

Kidney injury models

Male mice were administered 2mg/kg AAI dissolved in DMSO, or DMSO alone (control), by i.p. injection. For single nuclear RNA sequencing (snRNA-seq), control (TRE-h*KLF6*) and global h*KLF6* overexpression (h*KLF6*^{OE}) mice on a mixed background[1] were fed diet containing 200mg/kg DOX for one week prior to a single AAI injection, then euthanised 24 hours later. To generate a time course of AAI-induced injury, FVB/NJ mice (Jackson Labs) aged 9 weeks were given 1–4 injections 3 days apart, then euthanized 72 hours after the last injection. For PT fate tracing, *Slc34a1*-Cre^{ERT2};TdTomato mice were administered a series of 5 injections of 3mg tamoxifen dissolved in corn oil every other day. One week after the last tamoxifen injection, mice were administered two or three injections of 3mg/kg AAI 3 days apart, then euthanized 72 hours after the last injection. For AAI fibrosis studies, control and h*KLF6*^{TOE} mice aged 15-16 weeks old were injected every 3 days for 5 injections, followed by euthanasia after a further 2 weeks. For repeated low dose cisplatin (RLDC), male FVB/N mice aged 11 weeks were treated with normal saline (control) or 7mg/kg cisplatin once per week for 4 weeks, followed by euthanasia

one week after the last injection[3]. Male C57Bl/6 mice aged 12-15 weeks were subjected to unilateral ureteral obstruction (UUO), and UUO and contralateral control kidneys harvested after 3 days or 7 days as previously reported[4]. For all mice, blood and urine samples were collected under terminal anesthesia, followed by cardiovascular perfusion with PBS and removal of the kidneys for histological, RNA, and protein analyses.

Single nucleus RNA sequencing (snRNA-seq)

Mice were perfused with PBS, followed by harvesting of the kidneys and storage in RNAlater at -80°C. Nuclei were isolated and prepared for snRNA-seq with the 10X Chromium System (10X Genomics) as previously described, and sequenced using an Illumina NextSeq 550 sequencer[5]. Read depths were ~6,300 – 10,600 reads per cell. All samples underwent nuclear isolation, single nuclear preparation, and library preparation together at the same time, using the same kit, and were sequenced on the same lane, and thus comprised one single technical batch. Raw sequencing data were demultiplexed and aligned to a mouse pre-mRNA reference genome using Cell Ranger version 3.1.0 on SeaWulf, the High Performance Computing Cluster at Stony Brook University. Quality control (QC), dimensionality reduction, and clustering were performed using the R software package Seurat version 4.3[6]. Genes expressed in a minimum of three cells were retained. Nuclei expressing < 200 or > 6000 genes, or >10% mitochondrial genes were excluded[5,7]. A total of 34,146 out of 34,290 nuclei passed the QC parameters and were used for analysis. Clustering was performed using the first 30 principal components and a resolution of 0.8. Marker genes for each cluster were identified using the Seurat (version 4.3.0) “FindMarkers” function, with min.pct=0.1 and logfc.threshold=0.2. and clusters were then annotated using these marker genes by cross-matching with previously published datasets[8,9]. Differentially expressed genes between the same cluster in different samples were determined using the Seurat

“FindMarkers” function, with min.pct=0.1 and logfc.threshold=0.2. Genes were considered significant at adjusted p value of <0.05 (Bonferroni correction), but for pathway analysis only the top 300 genes were used for each condition. Cell cycle analysis was undertaken using the built-in “CellCycleScoring” function in Seurat. Pathway and gene ontology (GO) analyses were performed using clusterProfiler[10] with false discovery rate adjustment for multiple comparisons.

Single nuclear assay for transposase-accessible chromatin sequencing (snATAC-seq)

Nuclei were isolated and prepared from the same samples as for the snRNA-seq, using the 10X Chromium system (10X Genomics), according to manufacturer’s instructions. The snATAC-seq analysis was performed in R using the package ArchR 1.0.2[11]. Cells of low quality, defined as having fewer than 2500 fragments or a TSS enrichment score below 8, were excluded. Doublet removal was operated using embedded default parameters in ArchR. Prior to integrating the RNA matrix from Seurat, an embedding from snATAC-seq was obtained by adding a latent semantic index (LSI). After applying harmony to remove batch effects, a corresponding UMAP and clusters were generated. RNA integration was first performed with unconstrained conditions without a group list. The unconstrained integration results were plotted and visualized on the snATAC UMAP. The PT and non-PT clusters could be identified by the integration result, and the integration result was combined with snATAC-seq clusters as a group list. The constrained integration was then performed with the group list using the same method, and the gene integration matrix was incorporated into the ArchR project. To enhance the analysis, another LSI based on the gene integration matrix was constructed, and LSIs from snATAC-seq and snRNA-seq combined. The UMAP was finalized based on the combined LSI with harmony to remove batch effects and final cluster annotations were based on the constrained integration result.

Trajectory analysis and peak-to-gene linkages

Pseudotime trajectory analysis was conducted using the built-in functionality in ArchR. The trajectory originated from the healthy PTS2 cluster to the injury PT cluster. A gene expression heatmap was generated along the trajectory, showing the top 50 genes ranked by their variance. Peak-to-gene linkages were added using the built-in functionality in ArchR. Feature peaks were selected based on <0.05 false discovery rate (FDR) and >1.0 $\log_2$ fold change. Alternatively, we first regenerated the UMAP based on the RNA component alone, followed by trajectory analysis.

Reanalysis of published mouse scRNA-seq dataset

The cell count matrix was directly downloaded from GSE197266 and processed using R programming. For analysis, Seurat (version 4.2.1) along with SeuratObject (version 4.1.3) was utilized. Cisplatin injury samples and their corresponding controls were selected for further examination. Low-quality cells were filtered out based on the criteria 200<nFeature_RNA<7500 and percent.mt<5, resulting in 9,060 nuclei retained for downstream analysis. The dataset was normalized and scaled using SCTransform, followed by dimensionality reduction through principal component analysis (PCA). A total of 50 principal components were selected for clustering. To correct for batch effects, Harmony was applied. Clustering was performed using Seurat's default methods with a resolution parameter of 0.8, identifying 15 clusters. Differentially expressed genes (DEGs) for each cluster were generated and cross-referenced with marker genes reported in the paper. The PT cluster was then subsetted and reclustered using Seurat's default methods with a resolution of 0.8, resulting in 11 clusters. DEGs for these clusters were identified and compared with marker genes reported in the paper and validated through Kidney Interactive Transcriptomics from the Humphreys Lab.

Analysis of KPMP snRNA-seq dataset

The combined snRNA-seq h5Seurat file (doi.10.48698/mg7h-bc51)[12] was downloaded directly from the KPMP repository. KPMP patient metadata was used to add the enrollment category for each patient, and the dataset was then subsetted to remove the two diabetes mellitus resistant (DM-R) patients. The dataset therefore consisted of 25 healthy reference, 37 CKD, and 16 AKI samples. The metadata category "subclass.l2" was used to identify the clusters, and the differentially expressed genes between aPT or dPT and combined PT-S1/PT-S2/PT-S3 clusters were determined using the Seurat "FindMarkers" function, with min.pct=0.1 and logfc.threshold=0.1. Genes were considered significant at adjusted p value of <0.05 (Bonferroni correction).

Measurement of serum creatinine and urea nitrogen concentrations

Serum creatinine concentrations were measured using the isotope dilution liquid chromatography-tandem mass spectrometer at the University of Alabama at Birmingham (UAB) O'Brien Core Center. Serum urea nitrogen concentrations were measured by a colorimetric assay (Arbor Assays) according to the manufacturer's protocol[1].

Histology, immunofluorescence, and IHC

Formalin-fixed kidneys were processed, paraffin embedded, and sectioned at 5μm thickness. H&E and periodic acid Schiff (PAS) stains were performed according to standard protocols, and slides mounted using CytosealXYL permanent mounting medium. For immunofluorescent staining, slides were dewaxed, rehydrated and subject to heat induced epitope retrieval in 10mM citrate buffer, pH6 at 120°C for 10 minutes. Sections were blocked in 2% non-fat milk, followed

by incubation with rabbit anti-cytokeratin-20 (Abcam, ab97511), mouse anti-α-SMA (Sigma, A5228), rabbit anti-Vimentin (Cell Signaling, D21H3), or mouse anti-RPB1 conjugated with 594 fluorophore (Novus Biologicals, CTD 4H8) primary antibodies at 4°C overnight. After washing, sections were incubated with unlabeled secondary goat anti-rabbit (Jackson Immunoresearch, 111-005-144) followed by fluorescently labeled tertiary donkey anti-goat-568 (Life Technologies, A11057) or donkey anti-goat-647 (Life Technologies, A21447) antibodies, or with goat anti-mouse-488 (Life Technologies, A11029). PT brush borders were stained using FITC-conjugated Lotus lectin (Vector Labs) (mouse samples) or Phaseolus vulgaris Erythroagglutinin (PHA-E) (Vector Labs) (human samples), and nuclei were counterstained with Hoechst 33258 (Invitrogen). Slides were mounted in ProLong Gold Antifade mounting medium. For IHC for CXCL1, after dewaxing, rehydration and epitope retrieval, slides were incubated in 3% H_2O_2 at room temperature for 10 minutes, followed by blocking as above, and incubation with rabbit anti-CXCL1 (Life Technologies, PA5-86508) at 4°C overnight. After washing, sections were incubated with HRP-conjugated donkey anti-rabbit secondary antibody (Invitrogen, A16035) and DAB staining undertaken using the Betazoid DAB Chromogen kit (Biocare Medical). All slides were viewed using a Nikon Eclipse 90i microscope and DS-Qi2 camera. Images were analyzed and quantified using ImageJ 1.52i software (NIH) for percentage areas stained for Lotus lectin, cytokeratin-20, and α-SMA. For Lotus lectin and cytokeratin staining, one stitched image measuring ~1.5mm x 1.5mm was quantified for each mouse. For α-SMA, 8-10 20X images were quantified then the mean area calculated for each mouse, after excluding blood vessels by thresholding on the basis of intensity. RPB1+ nuclei were manually counted in 4-6 20X fields per mouse. For quantification of human samples, tubules were classified as PHA-E-positive, KRT-20-positive or double-positive, and the number of total (DAPI-positive) nuclei and $RPB1^{HI}$ nuclei were counted and $RPB1^{HI}$ nuclei calculated as a proportion of total nuclei. Where available, at

least 8 tubules per class were quantified in each of 3 separate 20X images from each sample. If fewer than 8 tubules were present in any one image, all were quantified.

In situ hybridization

In situ hybridization for h*KLF6* was undertaken using BaseScope (ACD Bio). Slides with 5μm-thick sections were processed through alcohols, followed by treatment with hydrogen peroxide at room temperature for 10 minutes, target retrieval at 98-102°C for 15 minutes, and treatment with protease III solution at 40°C for 15 minutes. After hybridization with a custom-made probe specific for human *KLF6* mRNA (with no cross-reactivity to mouse *Klf6*), the signal was amplified in a series of hybridization steps, according to manufacturer's instructions, before staining with fast red, followed by counterstaining with 20% solution of Gill's III hematoxylin. Slides were then dried and mounted in Vectashield permanent mounting medium, and examined using a Nikon Eclipse 90i microscope and DS-Qi2 camera.

Quantitative RT-PCR

RNA was extracted from kidney cortex using the RNeasy kit (Qiagen), DNase treated, and first strand cDNA synthesized from 1.25μg RNA using SuperScript IV VILO Mastermix (Life Technologies). Diluted cDNA was amplified in duplicate or triplicate using PowerUp SYBR qPCR Master Mix (Applied Biosystems) and specific primers for genes of interest, in an ABI QuantStudio 3 (Applied Biosystems). QuantStudio software was used to determine CT values using the second derivative method. Data were normalized to a reference gene (*Actb*) and presented as fold changes relative to RNA isolated from the control group using the $2^{-\Delta\Delta CT}$ method[13], or as or $2^{(-\Delta CT)}$ versus *Actb*. For measurement of mouse *Klf6* and human *KLF6*

expression, specific primers were used to detect gene expression from each species, and the $2^{(-\Delta CT)}$ versus *Actb* values were then combined to give total m*Klf6*+h*KLF6* expression.

Transient *POLR2A* knockdown

HK-2 cells were transfected with 10nM Trilencer-27 siRNA duplexes specific to *POLR2A* or Trilencer-27 universal scrambled control siRNA duplex (Origene), using SiTran2.0 transfection reagent (Origene), according to manufacturer's instructions. At the same time, cells were also treated with DMSO (control) or 25µM AAI for up to 48 hours. Cell numbers were assessed using CyQUANT Cell Proliferation Assay (Life Technologies). RNA was extracted using Trizol reagent, followed by DNase treatment, reverse transcription and qRT-PCR as for kidney cortex. For immunofluorescence analysis, cells were seeded onto glass coverslips. After transfection and treatment with DMSO/AAI, cells were fixed in cold 4% paraformaldehyde for 15 minutes, and permeabilized with 0.1% Triton X-100 for 10 minutes. After blocking in 10% donkey serum for 30 minutes at room temperature, cells were incubated with mouse anti-RPB1-594 conjugated antibody (4H8) (Novus Biologicals, NB200-598AF594), rabbit anti-Vimentin (Cell Signaling, D21H3), or rabbit anti-phospho-Histone H2A.X (Ser139) (γH2A.X) (Cell Signaling, 20E3) for 1 hour at room temperature, followed by goat anti-rabbit-488 (Life Technologies, A27034) for 1 hour at room temperature, and Hoechst 33258 (Invitrogen), then mounted in ProLong Gold Antifade mounting medium. For cell cycle analysis, single cells were fixed and permeabilized in 70% ethanol for >2 hours, followed by propidium iodide staining and analysis using a CytoFLEX flow cytometer (Beckman Coulter) at the Stony Brook University Flow Cytometry Research Core Facility. A minimum of 4000 cells were analyzed per replicate, and three replicates performed for each condition.

Bulk RNA-seq

Preparation of cDNA library and RNA-seq were performed by Novogene. Paired end fastq files were aligned to the human genome (hg38), quantified to the transcriptome (UCSC hg38 annotation) at the gene level, and differentially expressed genes (DEGs) between pairwise groups determined using the *limma* method via the BioJupies platform[14]. For SCR DMSO vs SCR AAI comparison, 1200 genes were upregulated and 760 genes were downregulated, and for SCR AAI vs siB AAI comparison, 906 genes were upregulated and 485 were downregulated, all at ≥2-fold or ≤0.5-fold and Benjamini-Hochberg adjusted $p<0.05$. DEGs were classified by gene length, and pathway and GO term analyses undertaken using ClusterProfiler[10].

Promoter mapping

KLF6 binding sites were obtained from ChIP-Seq data performed in HepG2 cells, deposited in the Gene Expression Omnibus database (accession no. GSE96355), and locations of binding sites were determined using the Genomic Regions Enrichment of Annotations Tool (GREAT)[15], “basal plus extension” approach, with a maximum extension of 10kb from any transcription start site (TSS). Binding site locations were mapped from human locations to mouse locations using the UCSC liftover tool (https://genome.ucsc.edu/cgi-bin/hgLiftOver). Locations of the binding sites relative to regions of open chromatin from mouse p2 nephron ATAC-seq (GSE124804)[16] were visualized using the R Bioconductor package Gviz.

Statistical Analysis

For mouse data, unpaired two-tailed Student’s t test with Holm-Sidak’s correction for multiple testing was used to compare data between two groups. One-way or two-way ANOVA with Sidak’s or Tukey’s corrections for multiple testing, respectively, were used to compare data

between more than two groups. The exact test used for each experiment is noted in the figure legends. Data are expressed as mean±SEM, and statistical significance assumed when $p<0.05$. Human *POLR2A* and *KLF6* gene expression data were extracted from previously reported microarrays[17,18], and European Renal cDNA Bank (ERCB) data of tubulointerstitial gene expression and GFR from healthy and diabetic nephropathy subjects, via Nephroseq. Pearson r correlation coefficients between continuous variables, and Mann-Whitney tests between groups were calculated. Data are expressed as box and whiskers, with box edges at 25th and 75th percentiles, whiskers at minimum and maximum, and line at the median. All analyses were undertaken using GraphPad Prism 9.

REFERENCES

1. Piret, SE, Guo, Y, Attallah, AA, Horne, SJ, Zollman, A, Owusu, D, Henein, J, Sidorenko, VS, Revelo, MP, Hato, T, Ma'ayan, A, He, JC, Mallipattu, SK: Kruppel-like factor 6-mediated loss of BCAA catabolism contributes to kidney injury in mice and humans. *Proc Natl Acad Sci U S A,* 118**:** e2024414118, 2021.
2. Mallipattu, SK, Horne, SJ, D'Agati, V, Narla, G, Liu, R, Frohman, MA, Dickman, K, Chen, EY, Ma'ayan, A, Bialkowska, AB, Ghaleb, AM, Nandan, MO, Jain, MK, Daehn, I, Chuang, PY, Yang, VW, He, JC: Kruppel-like factor 6 regulates mitochondrial function in the kidney. *J Clin Invest,* 125**:** 1347-1361, 2015.
3. Sharp, CN, Doll, MA, Megyesi, J, Oropilla, GB, Beverly, LJ, Siskind, LJ: Subclinical kidney injury induced by repeated cisplatin administration results in progressive chronic kidney disease. *Am J Physiol Renal Physiol,* 315**:** F161-F172, 2018.
4. Gu, X, Mallipattu, SK, Guo, Y, Revelo, MP, Pace, J, Miller, T, Gao, X, Jain, MK, Bialkowska, AB, Yang, VW, He, JC, Mei, C: The loss of Kruppel-like factor 15 in Foxd1(+) stromal cells exacerbates kidney fibrosis. *Kidney Int,* 92**:** 1178-1193, 2017.
5. Pace, JA, Bronstein, R, Guo, Y, Yang, Y, Estrada, CC, Gujarati, N, Salant, DJ, Haley, J, Bialkowska, AB, Yang, VW, He, JC, Mallipattu, SK: Podocyte-specific KLF4 is required to maintain parietal epithelial cell quiescence in the kidney. *Sci Adv,* 7**:** eabg6600, 2021.
6. Butler, A, Hoffman, P, Smibert, P, Papalexi, E, Satija, R: Integrating single-cell transcriptomic data across different conditions, technologies, and species. *Nat Biotechnol,* 36**:** 411-420, 2018.
7. Lu, YA, Liao, CT, Raybould, R, Talabani, B, Grigorieva, I, Szomolay, B, Bowen, T, Andrews, R, Taylor, PR, Fraser, D: Single-Nucleus RNA Sequencing Identifies New Classes of

Proximal Tubular Epithelial Cells in Kidney Fibrosis. *J Am Soc Nephrol,* 32**:** 2501-2516, 2021.

8. Kirita, Y, Wu, H, Uchimura, K, Wilson, PC, Humphreys, BD: Cell profiling of mouse acute kidney injury reveals conserved cellular responses to injury. *Proc Natl Acad Sci U S A,* 117**:** 15874-15883, 2020.

9. Wu, H, Kirita, Y, Donnelly, EL, Humphreys, BD: Advantages of Single-Nucleus over Single-Cell RNA Sequencing of Adult Kidney: Rare Cell Types and Novel Cell States Revealed in Fibrosis. *J Am Soc Nephrol,* 30**:** 23-32, 2019.

10. Yu, G, Wang, LG, Han, Y, He, QY: clusterProfiler: an R package for comparing biological themes among gene clusters. *OMICS,* 16**:** 284-287, 2012.

11. Granja, JM, Corces, MR, Pierce, SE, Bagdatli, ST, Choudhry, H, Chang, HY, Greenleaf, WJ: ArchR is a scalable software package for integrative single-cell chromatin accessibility analysis. *Nat Genet,* 53**:** 403-411, 2021.

12. Kidney Precision Medicine Project. Aggregated, clustered single-nucleus RNA-seq data used in the KPMP Atlas Explorer v1.5. 2024. https://doi.org/10.48698/mg7h-bc51

13. Pfaffl, MW: A new mathematical model for relative quantification in real-time RT-PCR. *Nucleic Acids Res,* 29**:** e45, 2001.

14. Torre, D, Lachmann, A, Ma'ayan, A: BioJupies: Automated Generation of Interactive Notebooks for RNA-Seq Data Analysis in the Cloud. *Cell Syst,* 7**:** 556-561 e553, 2018.

15. McLean, CY, Bristor, D, Hiller, M, Clarke, SL, Schaar, BT, Lowe, CB, Wenger, AM, Bejerano, G: GREAT improves functional interpretation of cis-regulatory regions. *Nat Biotechnol,* 28**:** 495-501, 2010.

16. Hilliard, S, Song, R, Liu, H, Chen, CH, Li, Y, Baddoo, M, Flemington, E, Wanek, A, Kolls, J, Saifudeen, Z, El-Dahr, SS: Defining the dynamic chromatin landscape of mouse nephron progenitors. *Biol Open,* 8, 2019.

17. Flechner, SM, Kurian, SM, Head, SR, Sharp, SM, Whisenant, TC, Zhang, J, Chismar, JD, Horvath, S, Mondala, T, Gilmartin, T, Cook, DJ, Kay, SA, Walker, JR, Salomon, DR: Kidney transplant rejection and tissue injury by gene profiling of biopsies and peripheral blood lymphocytes. *Am J Transplant,* 4**:** 1475-1489, 2004.

18. Nakagawa, S, Nishihara, K, Miyata, H, Shinke, H, Tomita, E, Kajiwara, M, Matsubara, T, Iehara, N, Igarashi, Y, Yamada, H, Fukatsu, A, Yanagita, M, Matsubara, K, Masuda, S: Molecular Markers of Tubulointerstitial Fibrosis and Tubular Cell Damage in Patients with Chronic Kidney Disease. *PLoS One,* 10**:** e0136994, 2015.

19. Balzer, MS, Doke, T, Yang, YW, Aldridge, DL, Hu, H, Mai, H, Mukhi, D, Ma, Z, Shrestha, R, Palmer, MB, Hunter, CA, Susztak, K: Single-cell analysis highlights differences in druggable pathways underlying adaptive or fibrotic kidney regeneration. *Nat Commun,* 13**:** 4018, 2022.

20. Chen, Z, Li, Y, Yuan, Y, Lai, K, Ye, K, Lin, Y, Lan, R, Chen, H, Xu, Y: Single-cell sequencing reveals homogeneity and heterogeneity of the cytopathological mechanisms in different etiology-induced AKI. *Cell Death Dis,* 14**:** 318, 2023.

SUPPLEMENTAL FIGURE LEGENDS

Supplemental Figure 1. Phenotype and quality control metrics in snRNA-seq samples. **(A)** Protocol for inducing AAI injury in h*KLF6*^{OE} mice for snRNA-seq analysis. **(B)** Serum creatinine in control and h*KLF6*^{OE} mice after one injection of AAI. **(C)** Histological analysis of kidneys used for snRNA-seq, using periodic acid Schiff (PAS) staining. Scale bars = 250μm (main images) and 50μm (magnified areas). **(D-E)** Quality control metrics for snRNA-seq samples pre- **(D)** and post- **(E)** filtering. The numbers of genes (nFeature_RNA), UMIs (nCount_RNA), and percentage of mitochondrial transcripts (percent.mt) are shown, along with the correlations between UMIs and percentage of mitochondrial transcripts, and between UMIs and genes, for each sample. A total of 34,290 cells passed the QC parameters and were used for analysis.

Supplemental Figure 2. Pathway and GO term analysis of DEGs in h*KLF6*^{OE} versus control mice for PT clusters. (A) Pathway analysis of upregulated genes in h*KLF6*^{OE} versus control PT clusters. **(B)** WikiPathways analysis of genes downregulated and upregulated in h*KLF6*^{OE} versus control PT clusters. **(C)** GO term analysis of upregulated genes in h*KLF6*^{OE} versus control PT clusters. GeneRatio = fraction of DEGs found in each gene set; p.adjust = false discovery rate.

Supplemental Figure 3. Expression of marker genes. (A) Expression of previously reported marker genes from “maladaptive PT” cluster[19]. **(B)** Expression of *Klf6* across all clusters and samples.

Supplemental Figure 4. Pathway and GO term analysis of cluster-specific DEGs in normal and injured PT clusters. The top 300 DEGs for each cluster were used. **(A)** Pathway analysis. **(B)** GO term analysis. GeneRatio = fraction of DEGs found in each gene set; p.adjust = false discovery rate. **(C)** DEGs contributing to upregulated KEGG pathways in Injured PT-B cells.

Supplemental Figure 5. KEGG pathway and cell cycle analyses. (A) KEGG analysis of DEGs in PT clusters from AAI-treated versus control mice. GeneRatio = fraction of DEGs found in each gene set; p.adjust = false discovery rate. **(B-C)** Cell cycle analysis **(B)** and quantification in PT clusters **(C)** .

Supplemental Figure 6. Identification of a *Klf6*/*Polr2a* injured PT cluster in a cisplatin scRNA-seq dataset. (A) UMAP of all cells from control and cisplatin samples. **(B)** Subclustering of PT cells from control and cisplatin samples. **(C)** Relative cell type abundance in control and cisplatin samples. Numbers above the bars indicate the total number of cells in each cluster. **(D)** Marker genes for the PT clusters. **(E,F)** WikiPathways **(E)** and GO biological processes **(F)** for the DEGs in the NewPT cluster versus other PT clusters. GeneRatio = fraction of DEGs found in each gene set; p.adjust = false discovery rate. Data reanalyzed from Chen et. al.[20].

Supplemental Figure 7. RPB1 expression in AAI, repeated low-dose cisplatin (RLDC) and unilateral ureteral obstruction (UUO) models. (A) Individual channels for RPB1 and vimentin coimmunofluorescence shown in Figure 2E. **(B)** Coimmunofluorescence for RPB1 and PT injury marker cytokeratin-20 (KRT-20) in mice treated with serial injections of AAI. **(C)** Fate tracing of PT by coimmunofluorescence for TdTomato and RPB1 in *Slc34a1*-Cre^{ERT2};TdTomato

mice treated with tamoxifen and AAI. All sections were counterstained with DAPI and Lotus lectin. Scale bars = 100µm (main images) or 20µm (vimentin-stained magnified areas) or 50µm (TdTomato and KRT-20-stained magnified areas).

Supplemental Figure 8. RPB1 expression in unilateral ureteral obstruction (UUO) and repeated low-dose cisplatin (RLDC) models. (A) Coimmunofluorescence for RPB1 and vimentin in contralateral control and UUO kidneys. **(B,C)** Coimmunofluorescence for RPB1 and vimentin **(B)** or KRT-20 **(C)** in mice treated with repeated low-dose cisplatin. All sections were counterstained with DAPI and Lotus lectin. Scale bars = 100µm (main images) or 20µm (vimentin-stained magnified areas) or 50µm (KRT-2 0-stained magnified areas).

Supplemental Figure 9. Knockdown of *POLR2A* in HK-2 cells. (A) mRNA expression of *POLR2A* in HK-2 cells treated with scrambled (SCR) or *POLR2A*-specific siRNA, with or without AAI treatment for 24 hours. Three different siRNAs were tested; si-B was selected for further experiments. **(B)** Representative histograms of cell cycle analysis using propidium iodide in HK-2 cells transfected with SCR or siB siRNAs, and treated with DMSO or AAI.

Supplemental Figure 10. Quality control metrics and cluster assignment in snATAC-seq samples. Distributions of the number of fragments **(A)**; fragment sizes **(B)**; transcription start site (TSS) enrichment **(C)**; and distance from TSS **(D)** in the snATAC-seq data. **(E)** Cell type specific marker RNA expression in the integrated snRNA-seq / snATAC-seq data. **(F)** Relative cell type abundance in the four samples. Numbers above the bars indicate the total number of cells in each cluster. **(G)** Motif enrichment for the injury cluster showed enrichment of injury-related transcription factors similar to those previously reported in AKI, such as AP1 members.

(H) Pathway analysis of the injury cluster showing similar active pathways to the snRNA-seq data.

Supplemental Figure 11. Potential role for KLF6 in regulation of *POLR2A* expression. (A-B) UMAP **(A)** and trajectory **(B)** based on RNA component of integrated data. **(C)** Expression of *Klf6* and *Polr2a* across pseudotime. **(D)** Mapping of open chromatin from published ATAC-seq in p2 mouse nephrons and KLF6 binding sites, with respect to the *Polr2a* locus. **(E)** Peak-to-gene analysis of open chromatin identified by snATAC-seq around the *Polr2a* locus in PT and injury clusters. **(F)** Transcription factor binding analysis of the *Polr2a* intron 1 injury open chromatin region (red box in part **(E)**) using the UCSC genome browser. **(G)** RNA levels of *Polr2a* in the injury cluster in control AAI (CtrlAAI) and h*KLF6*^{OE} AAI (OEAAI) samples. **(H)** *KLF6* and *POLR2A* mRNA expression in HK-2 cells stably expressing scrambled control (SCR) or *KLF6* shRNA (KD) constructs, and treated with DMSO (time 0) or 25μM AAI at 24 hours and 48 hours; n=3-4 per group. Unpaired Student's t-tests for each timepoint with FDR calculated using two-stage step-up (Benjamini, Krieger, and Yekutieli) correction for multiple testing.

Supplemental Figure 12. Tubular h*KLF6* overexpression alone does not cause kidney injury. Control (TRE-h*KLF6*) and h*KLF6*^{TOE} mice were given DOX in the diet continuously, starting at 6 weeks of age, for 18 weeks. **(A, B)** Serum creatinine **(A)** and urea nitrogen **(B)** concentrations in control and h*KLF6*^{TOE} mice after 18 weeks of DOX treatment. **(C)** Histological analysis using H&E and periodic acid-Schiff (PAS) stains. Scale bars = 250μm. **(D)** Examination of PT using Lotus lectin staining. Scale bars = 250μm. **(E)** Quantification of Lotus lectin staining. **(F)** Examination of h*KLF6* mRNA by *in situ* hybridization using BaseScope, with nuclei counterstained using hematoxylin. Scale bars = 100μm; arrowheads denote examples of

positive staining. **(G, H)** Kidney cortex mRNA expression of h*KLF6* **(G)** and m*Klf6* **(H)**. n=4-5 per group; unpaired Student's t-test.

Supplemental Figure 13. *Klf6* expression, kidney function, and branched-chain amino acid (BCAA) catabolism gene expression in control and h*KLF6*^{TOE} mice treated with AAI. **(A-C)** Kidney cortex mRNA expression of mouse (m) *Klf6* **(A)**, human (h) *KLF6* **(B)**, and combined m*Klf6*+h*KLF6* **(C)**. One-way ANOVA with Sidak's correction for multiple testing. **(D)** Correlation between total m*Klf6*+h*KLF6* expression and serum creatinine and urea nitrogen. Pearson r correlation with 2-tailed p value. **(E)** mRNA expression of genes encoding enzymes in the BCAA catabolic pathway. Unpaired Student's t-tests with FDR calculated using two-stage step-up (Benjamini, Krieger, and Yekutieli) correction for multiple testing.

Supplemental Figure 14. RPB1 immunofluorescence in control and h*KLF6*^{TOE} mice. (A, B) Immunofluorescent staining for RPB1 and vimentin (VIM), with counterstaining for Lotus lectin and DAPI. Scale bars = 100μm **(A)** or 20μm **(B)**. Areas in yellow boxes in **(A)** are shown in **(B)**. White dotted lines denote normal PT with low RPB1 expression; yellow dotted lines denote dedifferentiated PT with high RPB1 expression.

Supplemental Figure 15. RPB1 expression in CKD patients. Immunofluorescent staining for RPB1 with counterstaining for Phaseolus vulgaris Erythroagglutinin (PHA-E) and DAPI in patients with CKD and suspected (black labels) or confirmed (red labels) AA exposure. Scale bars = 100μm.

Supplemental Figure 16. RPB1 expression in control and diabetic kidney disease (DKD) patients. (A) Numbers of tubules per 20X field categorized into PHA-E-positive, KRT-20-positive, and double-positive for each sample. **(B)** Mean number of tubules per category for control and DKD patients. Two-way ANOVA with Sidak's multiple comparison's test. **(C)** Proportion of $RPB1^{HI}$ nuclei per tubule, categorized into PHA-E-positive, KRT-20-positive, and double-positive for each sample.

SUPPLEMENTAL FIGURE 1

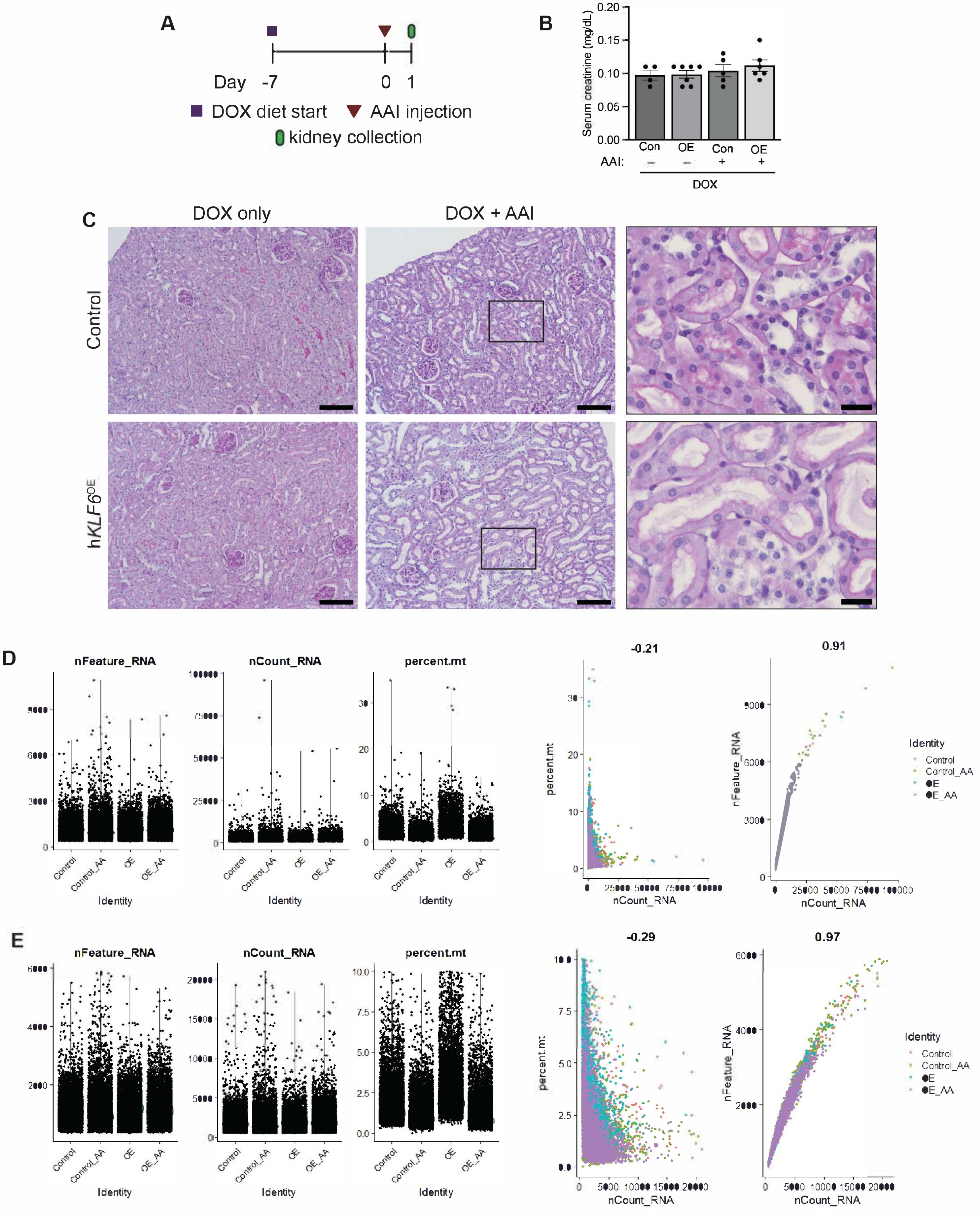

SUPPLEMENTAL FIGURE 2

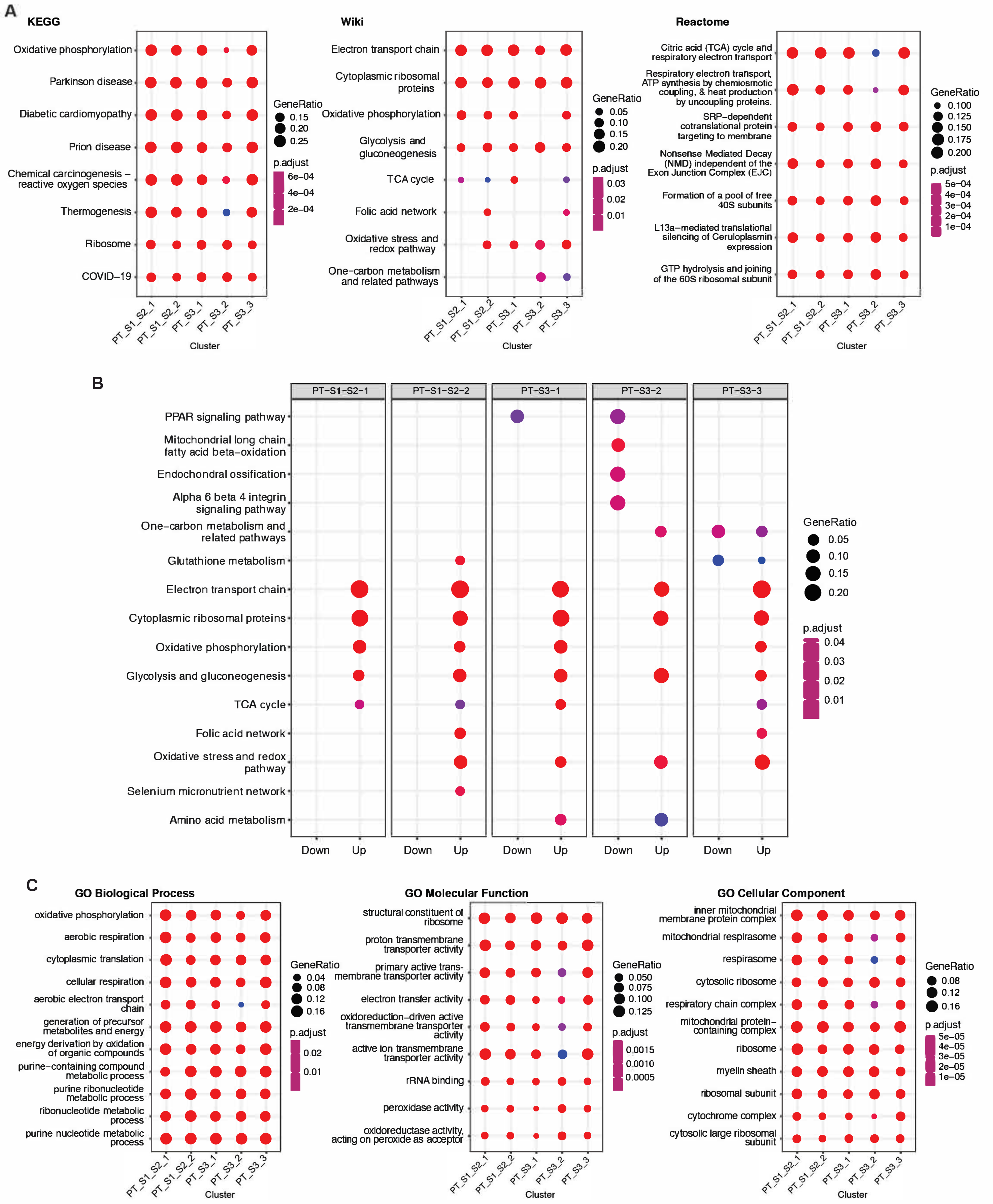

SUPPLEMENTAL FIGURE 3

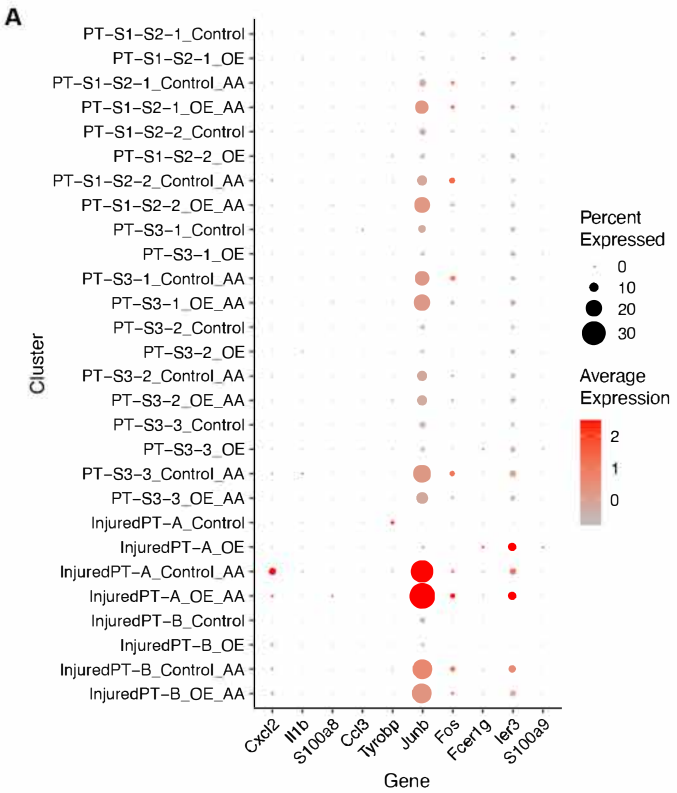

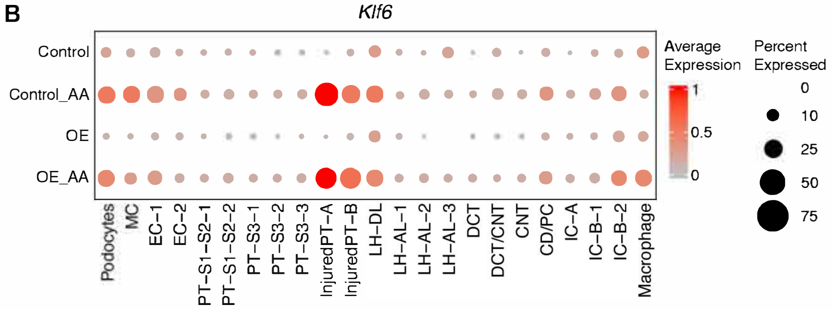

SUPPLEMENTAL FIGURE 4

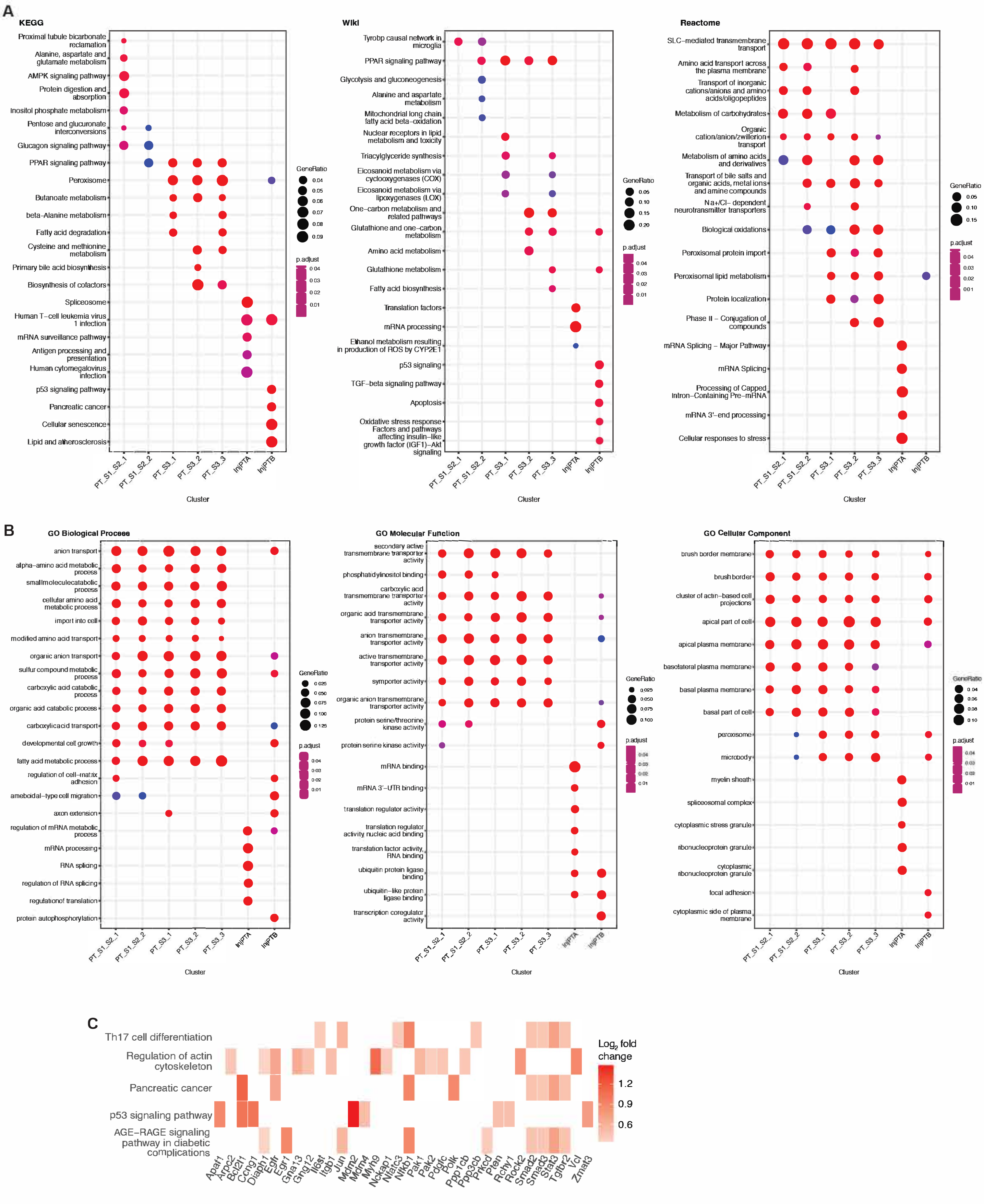

SUPPLEMENTAL FIGURE 5

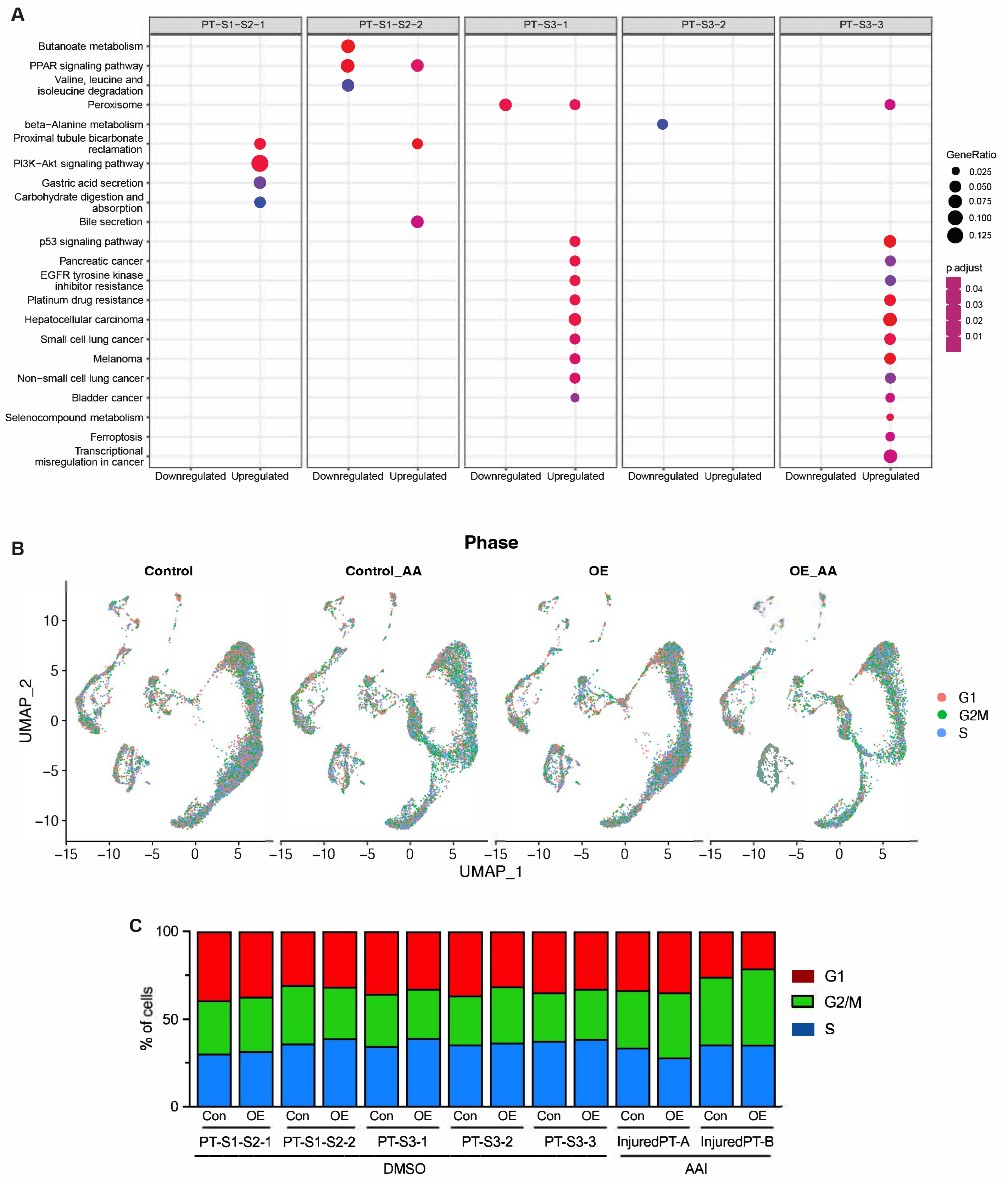

SUPPLEMENTAL FIGURE 6

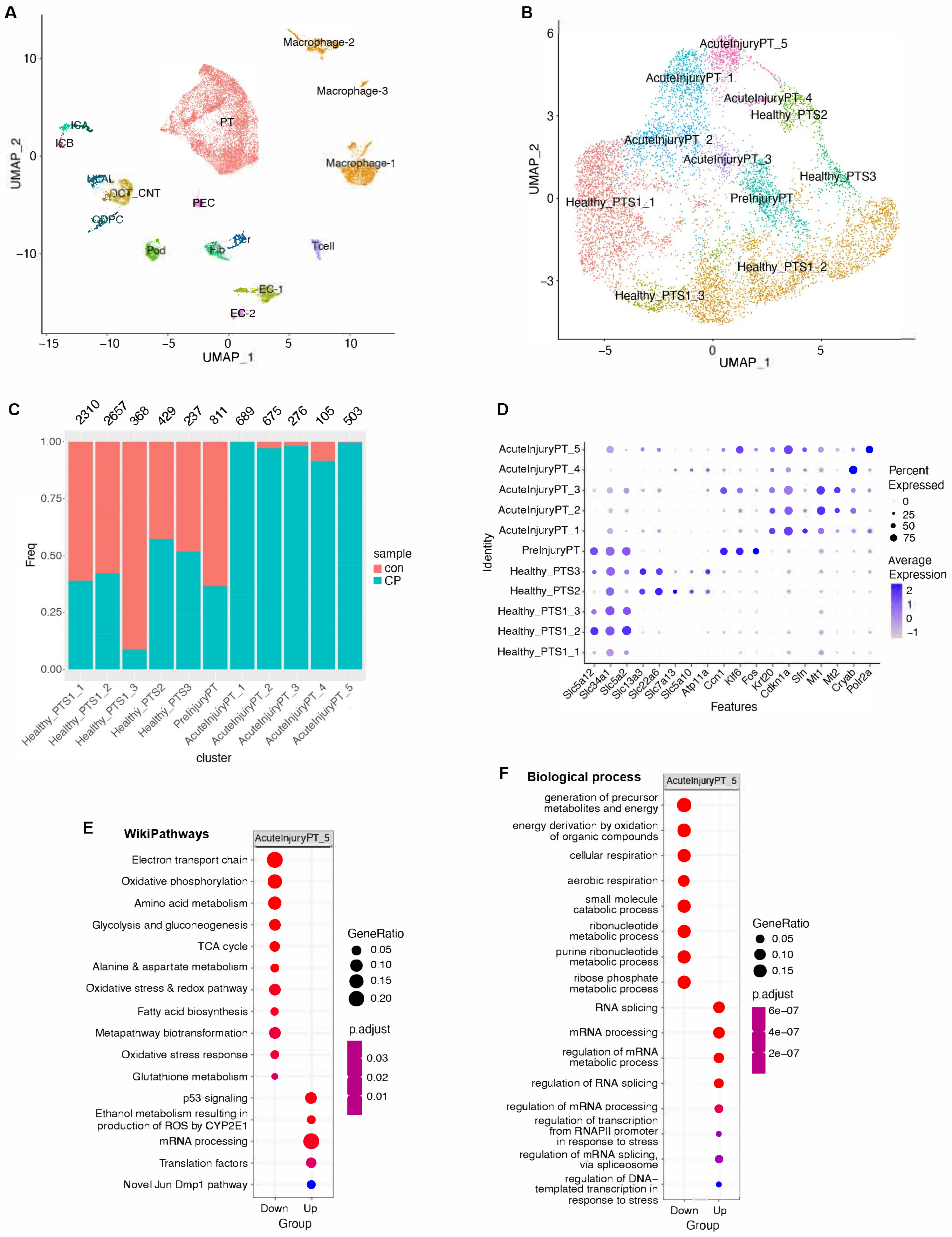

SUPPLEMENTAL FIGURE 7

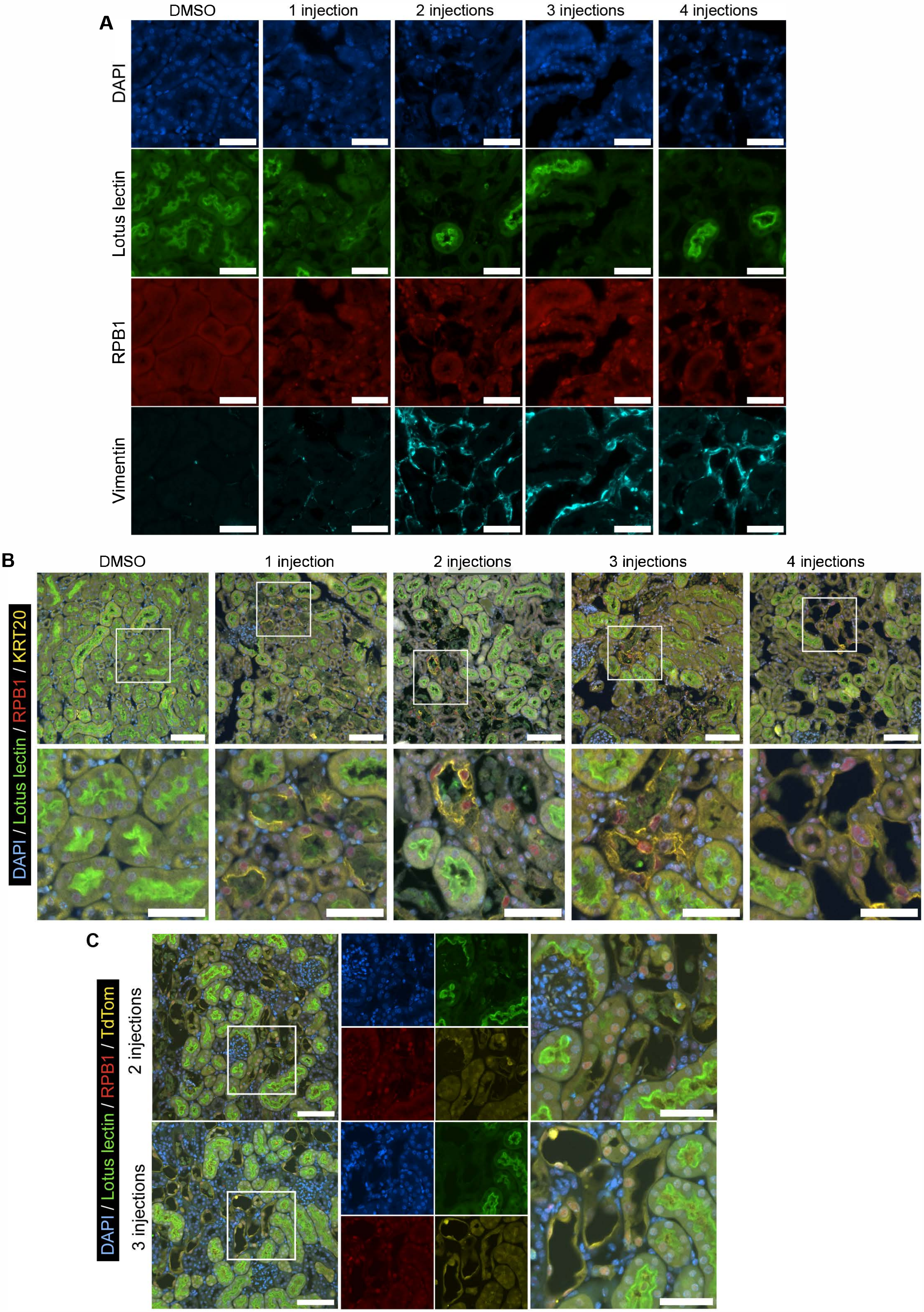

SUPPLEMENTAL FIGURE 8

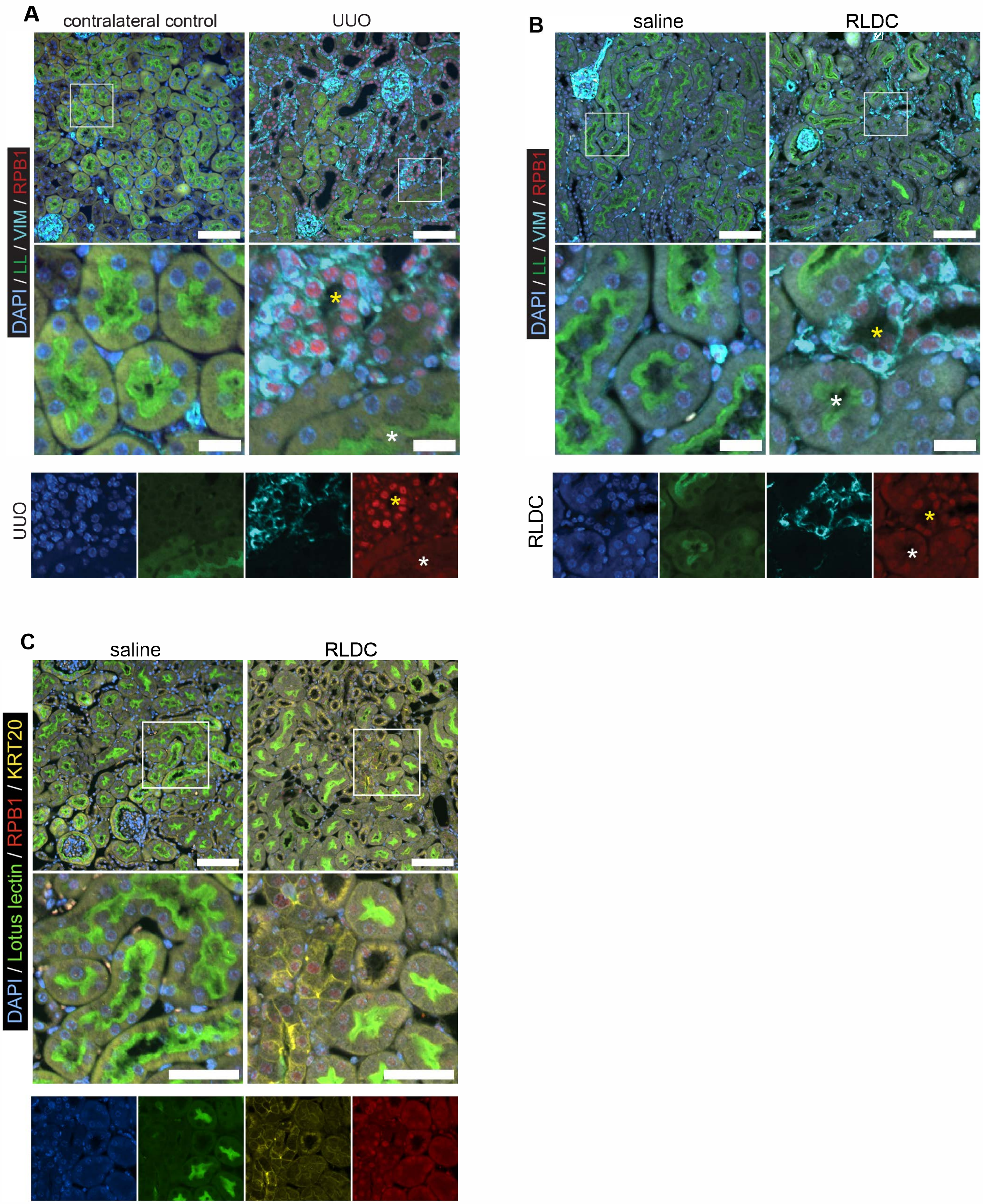

SUPPLEMENTAL FIGURE 9

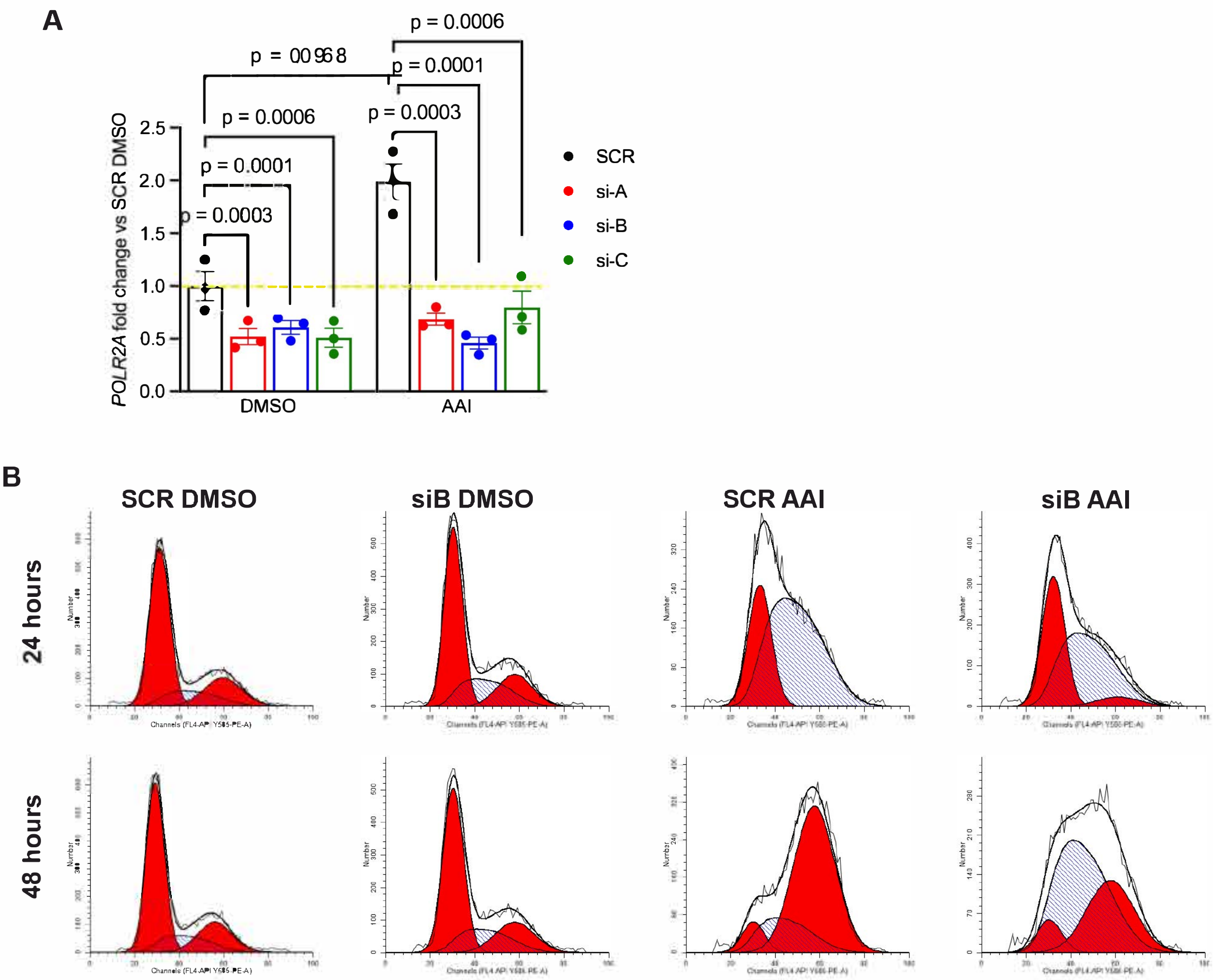

SUPPLEMENTAL FIGURE 10

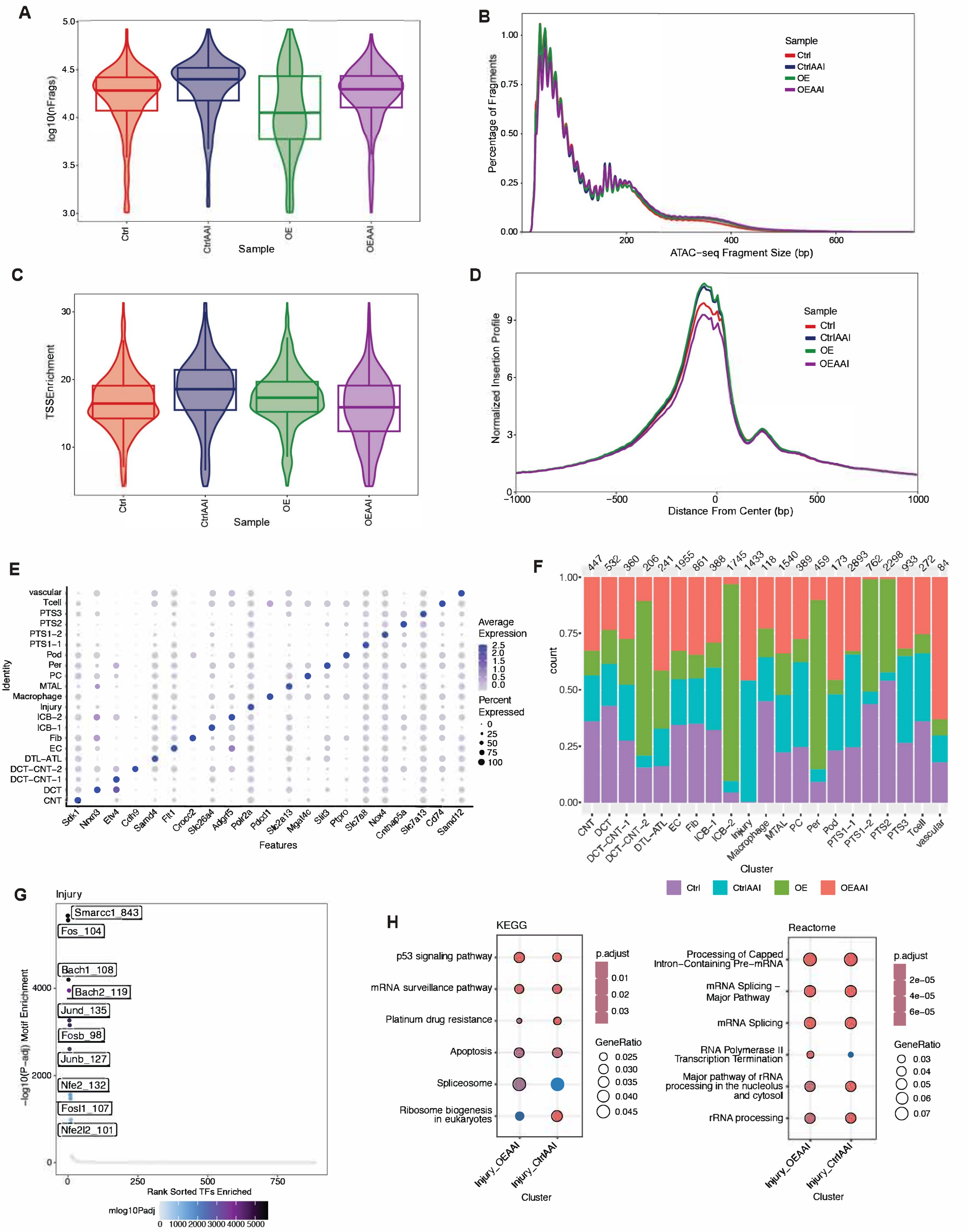

SUPPLEMENTAL FIGURE 11

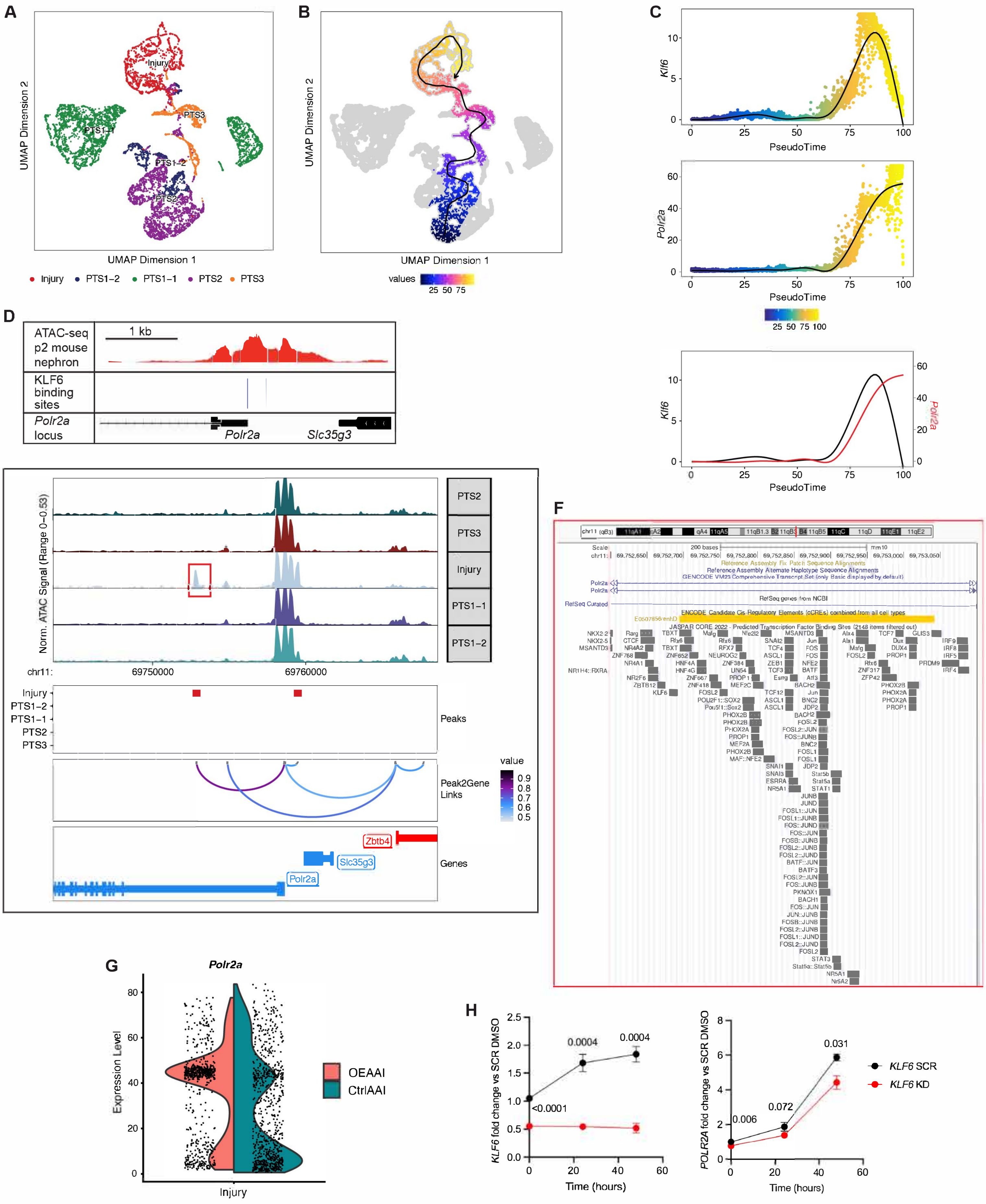

SUPPLEMENTAL FIGURE 12

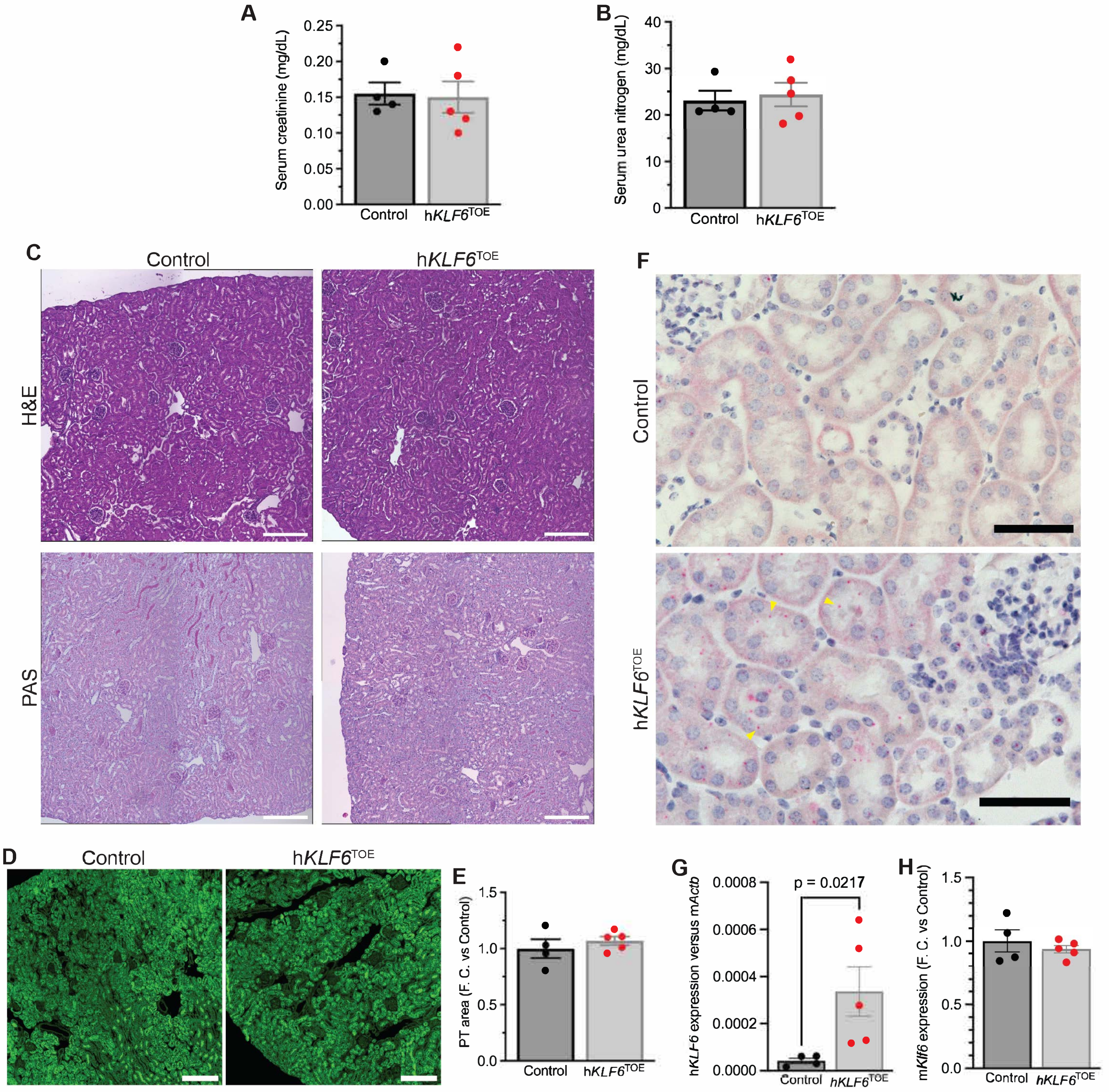

SUPPLEMENTAL FIGURE 13

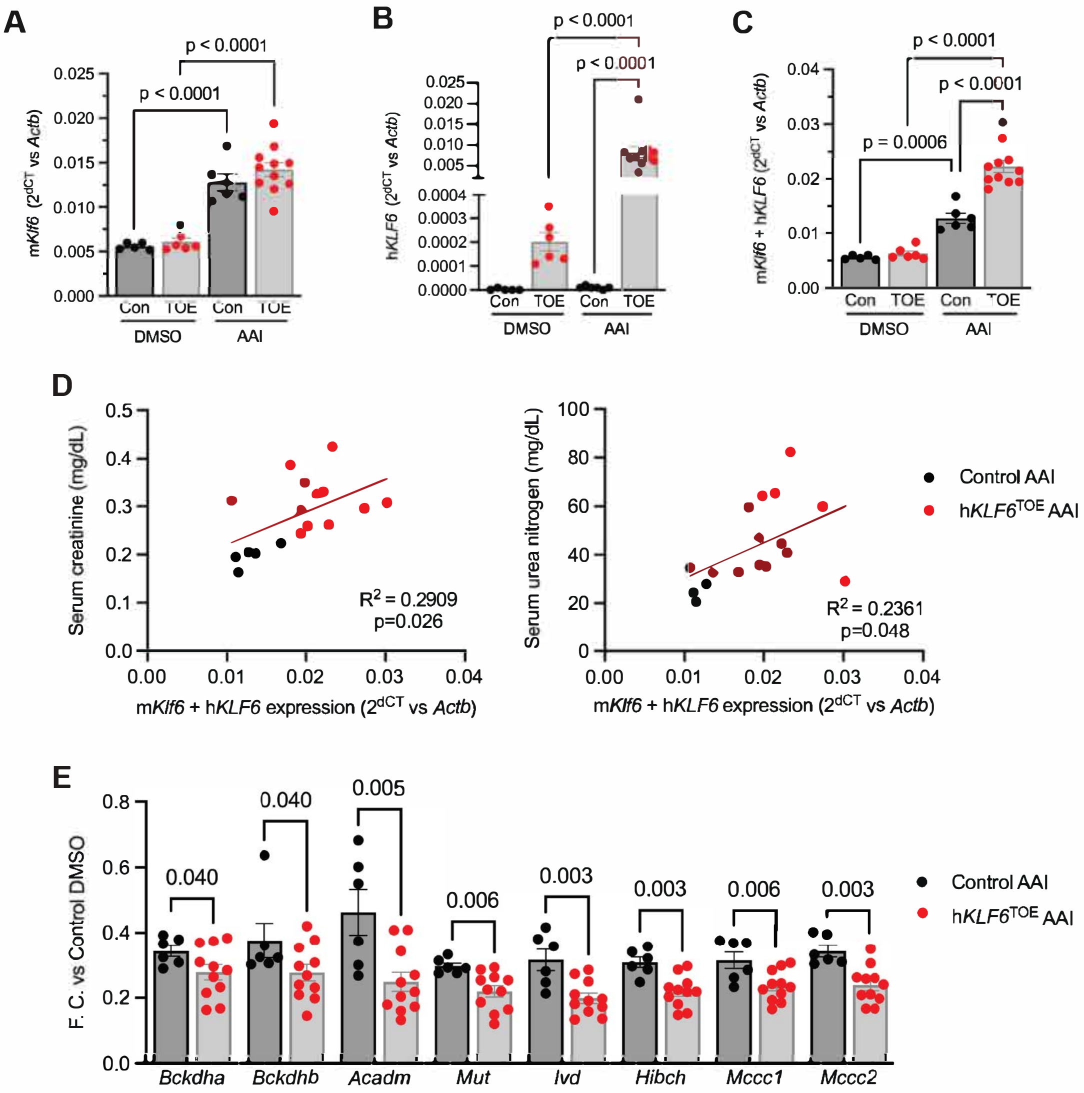

SUPPLEMENTAL FIGURE 14

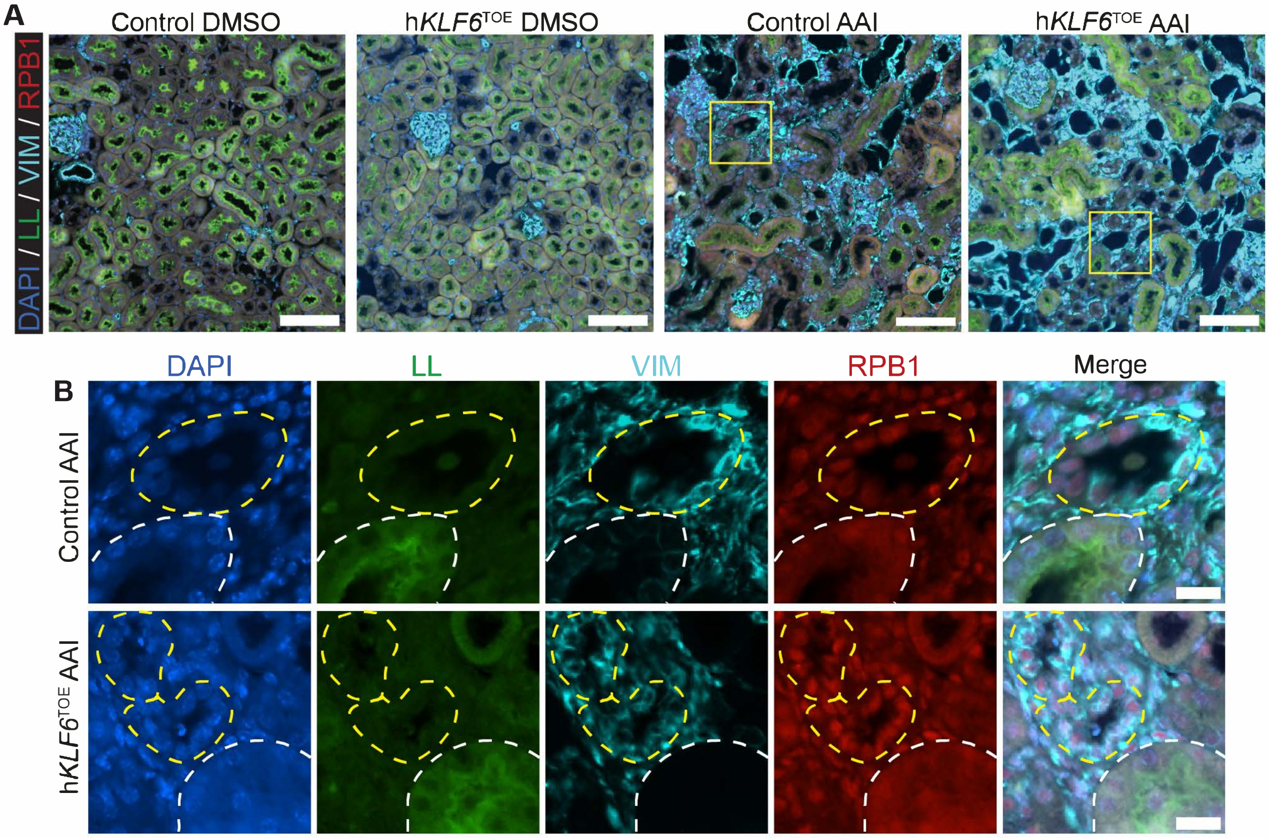

SUPPLEMENTAL FIGURE 15

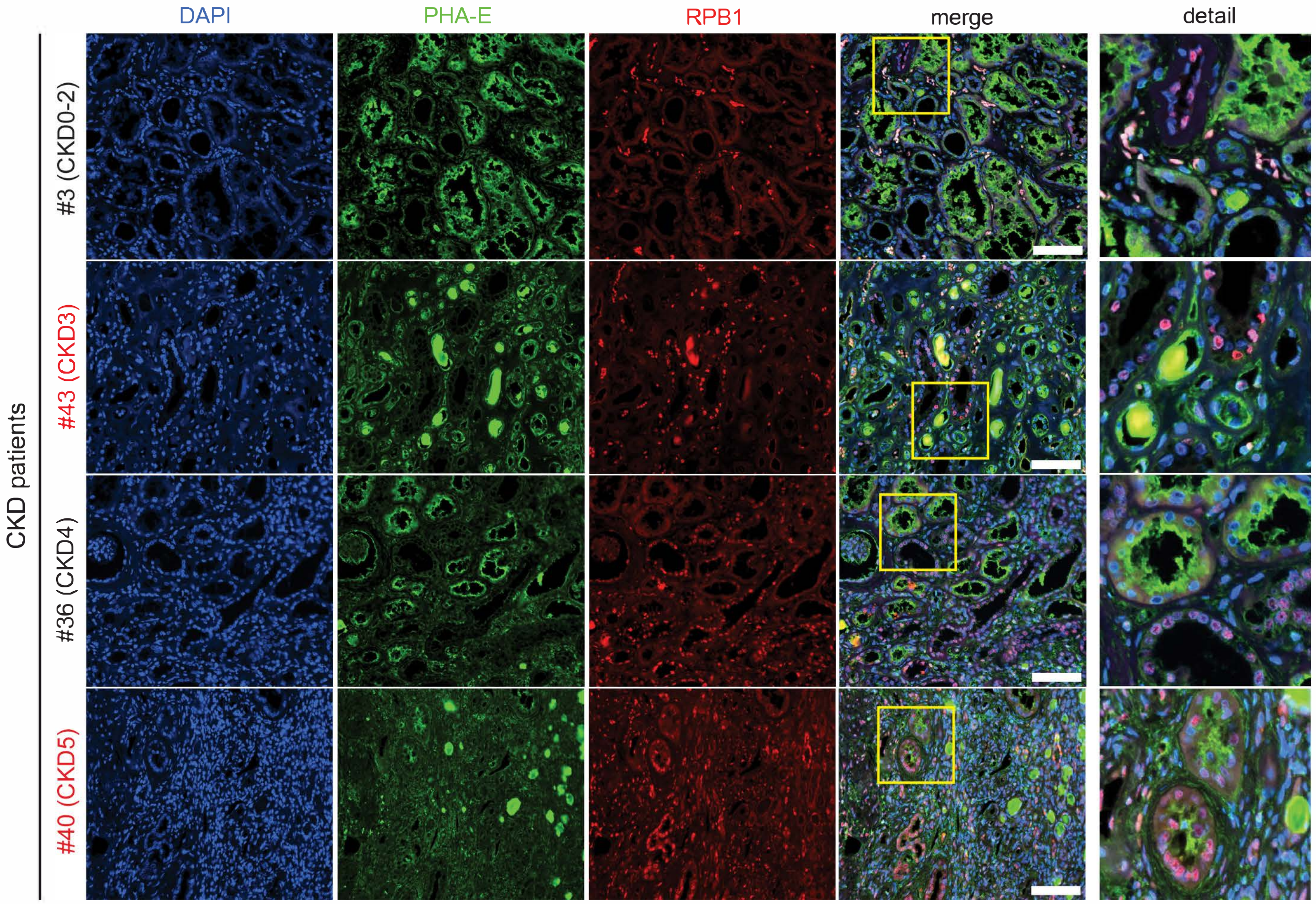

SUPPLEMENTAL FIGURE 16

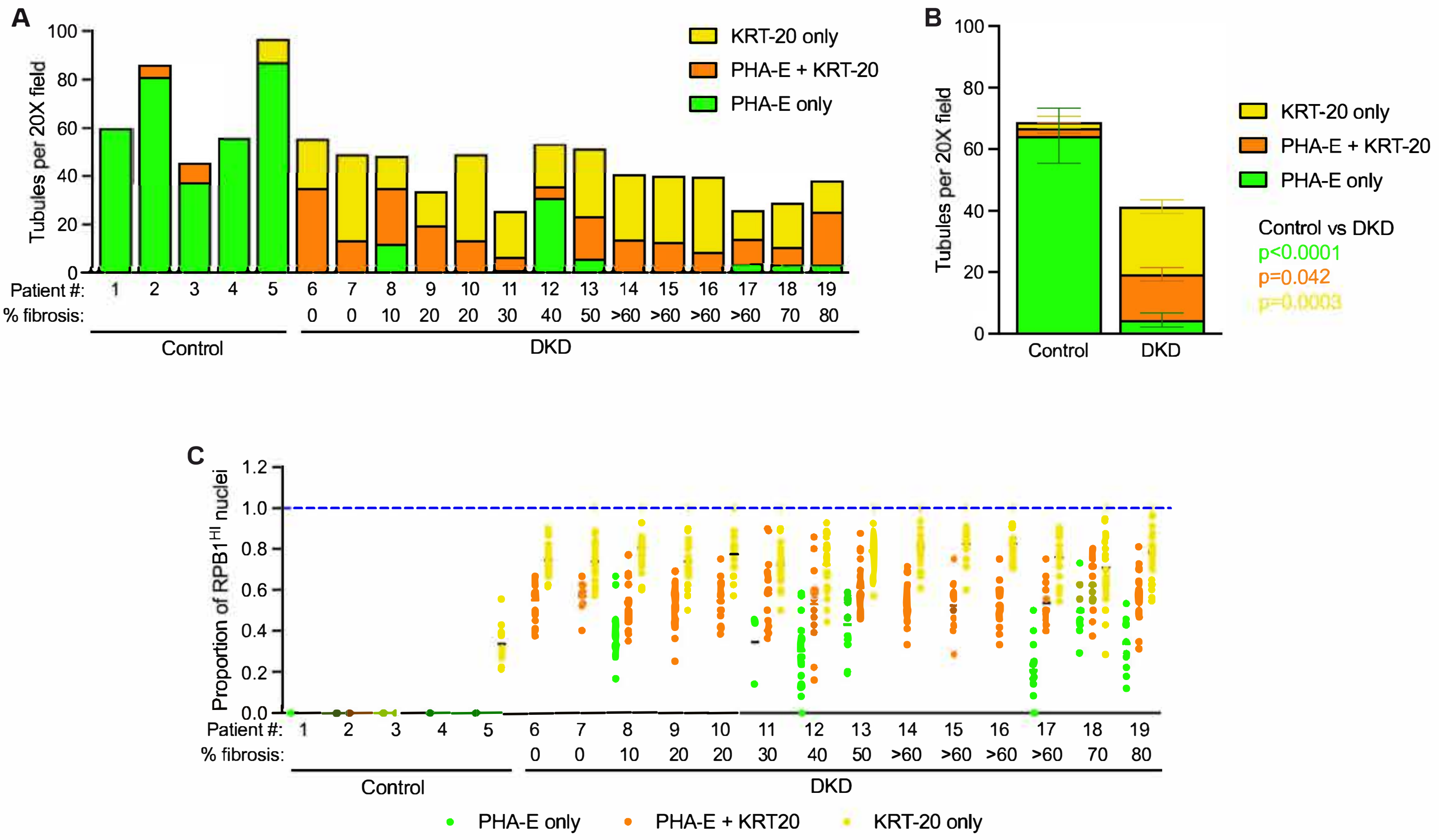

Supplement: SUPPLEMENTARY MATERIAL [file jasn-36-1914-s003.pdf]
